# Negative Impacts of Arsenic on Plants and Mitigation Strategies

**DOI:** 10.3390/plants12091815

**Published:** 2023-04-28

**Authors:** Dwaipayan Sinha, Soumi Datta, Reema Mishra, Preeti Agarwal, Tripti Kumari, Sherif Babatunde Adeyemi, Arun Kumar Maurya, Sharmistha Ganguly, Usman Atique, Sanchita Seal, Laxmi Kumari Gupta, Shahana Chowdhury, Jen-Tsung Chen

**Affiliations:** 1 Department of Botany, Government General Degree College, Mohanpur 721436, Paschim Medinipur, West Bengal, India; 2 Bioactive Natural Product Laboratory, School of Interdisciplinary Sciences and Technology, Jamia Hamdard, Hamdard Nagar, New Delhi 110062, India; 3 Department of Botany, Gargi College, University of Delhi, New Delhi 110049, India; 4 Department of Chemistry, Gargi College, University of Delhi, New Delhi 110049, India; 5 Ethnobotany/Phytomedicine Laboratory, Department of Plant Biology, Faculty of Life Sciences, University of Ilorin, Ilorin PMB 1515, Kwara State, Nigeria; 6 Department of Botany, Multanimal Modi College, Modinagar, Ghaziabad 201204, Uttar Pradesh, India; 7 University Department of Botany, Ranchi University, Ranchi 834008, Jharkhand, India; 8 Department of Bioscience and Biotechnology, College of Biological Systems, Chungnam National University, Daejeon 34134, Republic of Korea; 9 Department of Botany, Polba Mahavidyalaya, Polba 712148, West Bengal, India; 10 Bioprocess Development Laboratory, Department of Biotechnology, National Institute of Technology Warangal, Warangal 506004, Telangana, India; 11 Department of Biotechnology, Faculty of Engineering Sciences, German University Bangladesh, TNT Road, Telipara, Chandona Chowrasta, Gazipur 1702, Bangladesh; 12 Department of Life Sciences, National University of Kaohsiung, Kaohsiung 811, Taiwan

**Keywords:** arsenic, detoxification, food security, health risk, mitigation, sustainable development

## Abstract

Arsenic (As) is a metalloid prevalent mainly in soil and water. The presence of As above permissible levels becomes toxic and detrimental to living organisms, therefore, making it a significant global concern. Humans can absorb As through drinking polluted water and consuming As-contaminated food material grown in soil having As problems. Since human beings are mobile organisms, they can use clean uncontaminated water and food found through various channels or switch from an As-contaminated area to a clean area; but plants are sessile and obtain As along with essential minerals and water through roots that make them more susceptible to arsenic poisoning and consequent stress. Arsenic and phosphorus have many similarities in terms of their physical and chemical characteristics, and they commonly compete to cause physiological anomalies in biological systems that contribute to further stress. Initial indicators of arsenic’s propensity to induce toxicity in plants are a decrease in yield and a loss in plant biomass. This is accompanied by considerable physiological alterations; including instant oxidative surge; followed by essential biomolecule oxidation. These variables ultimately result in cell permeability and an electrolyte imbalance. In addition, arsenic disturbs the nucleic acids, the transcription process, and the essential enzymes engaged with the plant system’s primary metabolic pathways. To lessen As absorption by plants, a variety of mitigation strategies have been proposed which include agronomic practices, plant breeding, genetic manipulation, computer-aided modeling, biochemical techniques, and the altering of human approaches regarding consumption and pollution, and in these ways, increased awareness may be generated. These mitigation strategies will further help in ensuring good health, food security, and environmental sustainability. This article summarises the nature of the impact of arsenic on plants, the physio-biochemical mechanisms evolved to cope with As stress, and the mitigation measures that can be employed to eliminate the negative effects of As.

## 1. Introduction

Potentially toxic elements (PTEs) are of primary environmental concern worldwide causing pollution of water, air, and soil [1], and proving harmful to both flora and fauna, including humans [2]. Potentially toxic element uptake in plants is related to soil contamination, which may be due to natural or man-made reasons where the concentration of contaminants surpasses the permissible limits [3]. The natural reasons for PTEs pollution vary from the geochemical composition of source rocks to several soil formation mechanisms like volcanic eruptions, sedimentation, and weathering [4]. In contrast, man-made activities include smelting and mining operations, inadequate industrial and domestic waste disposal, industrial operation spills, misuse of agrochemicals [5], military practices, and oil and gas production [6]. The total PTEs load in the soil is equivalent to the cumulative inputs from both the sources (natural and man-made) subtracted from the total loss that occurred due to leaching, soil erosion, plant uptake, and volatilization of gaseous forms [7]. Various techniques like isotope ratio analysis, multivariate statistical analysis, geographic information system-based data, and target and reference elements enrichment factors are used to differentiate between the two types of PTEs sources [8].

The potentially lethal PTEs responsible for the contamination of soil are arsenic (As), zinc (Zn), lead (Pb), selenium (Se), cadmium (Cd), vanadium (V), cobalt (Co), silver (Ag), manganese (Mn), copper (Cu), mercury (Hg), antimony (Sb), chromium (Cr), barium (Ba), molybdenum (Mo), titanium (Ti), nickel (Ni), and tin (Sn) [9]. These PTEs are extremely harmful to living organisms (including humans) as they enter human food by various pathways [10] and contaminate the food chain [11]. The chemical characteristics of PTEs are accountable for their bioavailability and toxicity to human health [12].

It was reported that 16.1% of the soil samples collected during a study in China exceeded the endorsed standards of the Chinese Ministry of Environmental Protection, thus leading to compromised food quality and the jeopardizing of human health, thus indicating the disastrous effect of soil pollution [13]. In China, a survey reported as the Third National Survey on Causes of Death acknowledged 13 regions as cancer hotspots, namely Anhui Province (Yingdong District of Xinyang), Henan Province (Shenqui and Jun counties), and Hubei Province (Yingcheng), unveiling higher deaths caused by cancer than compared to the national average [14]. The WHO, in 2016 [15] reported the death of approximately 12.6 million people globally due to various types of diseases caused by polluted soil. Similarly, in Bangladesh, record-breaking deaths occurred due to PTEs; where, out of the total 125 million, 35–77 million people have been impacted by As contagion [16]. Lead is yet another major PTE element responsible for the death of millions of people worldwide. In 2015, the WHO along with the Institute for Health Metrics and Evaluation data reported damages of 9.3 million disability-adjusted life years and the loss of 494,550 lives [17]. Lead-polluted soil led to the poisoning and death of many of these, especially children in Senegal, Nigeria, and several other nations [18]; and caused mental retardation due to increased blood Pb levels in children globally [19]. Therefore, there is a need to identify and standardize soil remediation techniques to eliminate increased risks to humans caused by polluted soils.

Potentially toxic element contamination has resulted in enhanced land degradation and reduced productivity; therefore, soil remediation is the need of the hour and a matter of great interest in the scientific community [20]. To overcome these PTEs-associated contaminations, several soil remediation techniques have been applied both in situ and ex situ to increase fertile land for agriculture [21]. Technologies in situ include immobilization solidification/stabilization [22], soil washing/flushing [23], biological i.e., microbial treatments, and phytoremediation; while ex situ procedures are vitrification landfilling, soil washing [24], and bioreactors [25].

However, all soil remediation techniques have their own merits and demerits; and varying operational principles, cost strategies, and efficacies among the field and laboratory methods [26]. For commercial viability, soil alteration methods are applied to restrain PTEs contamination [27], therefore, it is imperative to choose proper agents for better pocket-friendly PTEs remediation methodologies and achieve principles of “green and sustainable remediation” owing to shorter life cycle ecosystem footprints [28]. This review is an attempt to highlight As as an environmental contaminant. Efforts have been made to give an in-depth overview of the sources of As, its biogeochemistry, and speciation, and ultimately to elaborate on the different effects of As on the animal system at large. The review culminates in the mitigation strategies of As in a sustainable way.

## 2. Methodology

The objective of the review was to integrate the available and relevant scientific information about As as a contaminant, its impacts, perception, and tolerance by plant systems, and mitigation measures in general adopted by human beings as well as man-made strategies that augment plants to cope with As stress. The review has been broadly divided into three parts. The first part deals with an introduction to As as a contaminant, its chemical nature, its toxicity, speciation, and factors responsible for speciation. The second part of the article is the most important and consists of the effects of As on plant systems in terms of a detailed description of morphological, and physio-biochemical effects. A connecting link between the first and second part has also been drawn which highlights As perception and uptake in the plant body. The third part of the article deals with the mechanisms to alleviate the toxicity of As in plants. This part also highlights the mitigation strategies of As toxicity.

This review has been meticulously designed in an integrative mode with a large volume of information pooled from the web. The information was pooled through previously planned searches with specific keywords. For example, the concept of potentially toxic elements (PTEs) has been dealt with in the initial portion of the article using keywords such as ‘potential toxic elements’, ‘worldwide PTE’ etc. Information about the speciation of As was pooled from the web using keywords such as ‘chemical speciation of arsenic in soil’ and ‘inter-conversion between forms of arsenic’. The effect of As on the plant system was searched in detail with a wide array of keywords, with a few important of these being ‘biochemical effect of arsenic on plants’, ‘arsenic and plant morphological changes’ ‘arsenic toxicity and plant oxidative stress’. The role and interaction of Nitric oxide (NO) with As were searched with key words such as ‘NO + As tolerance in Plants’, ‘NO + Arsenic effects in plants’, or ‘mitigation As stress by NO’. The papers on the mitigation strategies were sorted based on keywords like ‘conventional approach on mitigation strategies of arsenic’, ‘biotechnological approaches on mitigation strategies of arsenic’, and so on.

The internet was used as a search platform and review and research papers were downloaded from conventional search engines such as Google and Google Scholar. In addition, specialized searches for review and research papers were also performed in PubMed, Web of Science, and Scopus databases. Titles of the papers were shortlisted and very old papers were avoided with the exception of whether they were pioneer work or whether they imparted important information on the chemical and biochemical domain of As. The papers were segregated based on their themes and the major information was pooled to compose the manuscript. The results are elaborated in detail under the following heads.

## 3. Arsenic, a Dominant Environmental Contaminant

Arsenic is one of the most potent non-metalloid PTE environmental contaminants among all others [29] found that are toxic to plants; As with its associated molecules, is included in Group 1 of human carcinogens [30] by the International Agency for Research on Cancer (IARC) and the United States Environmental Protection Agency (US EPA). Therefore, As contamination is a global threat to the ecosystem as it is an extremely toxic element [31], is carcinogenic [32], and causes major health issues. Among 20 important hazardous substances mentioned by the Agency for Toxic Substances and Disease Registry (ATSDR), As is the top-ranked [33]. Table 1 illustrates the physico-chemical properties of As. 

Arsenic is regarded as non-essential in plants and other living creatures. Plants exposed to As at a minimum concentration are capable of causing biochemical, molecular, physiological, and morphological alterations [36]. The studies on soil-plant interaction have revealed the concept of ‘speciation of plants’ to various forms of As exposure, i.e., each plant species has a unique mechanism of uptake, toxicity, and accumulation of different types of As (arsenite, arsenate, As-III, and As-V).

There are various ways to accumulate As in the ecosystem, either by natural mechanisms like volcanic explosions or weathering of As-enriched minerals in underground core areas or by man-made activities like undue use of As-based chemicals in underground water [37], fertilizers in agriculture, mining, and smelting [38]. The presence of As is a common occurrence under the earth as out of the total mineral content, As and its compounds (arsenate) form 60%, sulfur salts, and sulfides 20%, and the rest comprises oxides, silicates, arsenides, arsenites and other forms of As [39]. Globally, it is estimated that one out of sixty persons live in regions with underground concentrations of As in water 50 µg L^−1^ or higher [40]. In Southeast Asia and the Indian subcontinent, As-contaminated food and water have poisoned millions of humans in recent years [41]. In soil and water, As is observed in four distinct oxidation states (inorganic and organic), namely As-III, As-V, As-III, and As-0 [42], while inorganic forms are two, viz. Arsenic-V (arsenate) and As-III (arsenite), and are most likely to be absorbed by plant roots [43]. As-III is more toxic [43], and it can be easily converted from As-V when absorbed by cells; however, both are harmful to plant metabolism but through separate mechanisms. The former exists in reduced condition, while the latter is under oxidization [44]. In *Triticum aestivum,* greater toxicity is reported in the organic form of As like dimethyl arsenate (DMA) than in As-V [45].

The origination of reactive oxygen species (ROS) at the subcellular level, like hydroxyl radical (OH), superoxide radical (O_2_^−^), and hydrogen peroxide (H_2_O_2_) is the most perilous biochemical effect of contamination by As [46]. Plant metabolism is severely affected by ROS as these cause irreparable harm to DNA, lipids, proteins, carbohydrates, and macro-molecules [47]. Sometimes, the transformation of As-V to AS-III is associated with ROS generation as well [48]. The harm caused by oxidative destruction due to ROS could be minimized by improving the capacity of the antioxidant enzymes [49].

In aerobic soils, arsenate is the predominant form and is equivalent to phosphate; and it is known to compete with comparable uptake transporters in the plant root plasma-lemma [50]. The root is the first tissue exposed to As contamination, where the non-essential metalloid hinders the proliferation and extension of the root. Once the As is translocated to the shoot, it impedes the growth of the plant by retarding the accretion and extension of biomass and inhibiting the reproducibility of the plant, yield loss, fertility, and fruit production [51]. In plants, the As uptake depends mainly on two factors, firstly, its total concentration, and secondly, on As-soil speciation (depends on As naturally available concentration in soil, which may be exchangeable) [52]. It was also reported that the transporter proteins in plants are directed by the level of As gradient among the cells and growth media when As penetrates the plant system as an inorganic species (As-III or As-V) [53].

Arsenic in a very high quantity interferes with crucial metabolic processes and results in mortality [41]; however, plants are mostly equipped with mechanisms to overcome such As-contaminated roots, whereas the amount of As translocated to the shoots and other plant parts is genotype dependent.

Arsenic toxicity disrupts numerous physiological activities like damaging the cellular membranes accompanied by enhanced malondialdehyde (MDA, a by-product of lipid peroxidation) production, inducing anti-oxidant defense mechanisms and electrolyte leaking [54]. It also leads to changes in the bio-geochemical behavior of As-contaminated soil [55]; like volatilization, biological transformations, oxidation-reduction, precipitation/dissolution, As-ligand complex formation, diversification of plant species, and sorption-desorption.

However, in plants, exact information about the specific As uptake transporters is not adequate to reach any irrefutable conclusion [56]. Phosphorous and As are analogous; both use the same transporters to transfer P and As to the root cell’s plasma membrane [57]. In some plant species, suppression of arsenate uptake/like-affinity phosphate helps reduce the influx of arsenate and thus increases tolerance towards arsenate; however, such plants can still accumulate a higher level of As over a period indicating a gamut of specific detoxification methodologies [58].

## 4. Classification of Arsenic from the Biological and Toxicological Point of View

Arsenic is highly toxic and a potential carcinogen. Environmental pollution with As is a global concern due to its harmful physiological effects on living systems. The presence of As, especially in soil and groundwater [59] directly exposes plants to significant risk [60,61]. It accumulates in different parts of edible plants growing in contaminated soils and water, which poses a grave threat when consumed by higher-order consumers in the food chain [62]. Arsenic can exist in many chemical forms—inorganic (exhibiting variable oxidation states) and/or organic in soil and water environments. Each chemical species has distinct physical and chemical characteristics, resulting in varying bio-geochemical properties like mobility, solubility, bioavailability, and toxicity [61]. Therefore, understanding As chemical speciation in soil-plant systems and its effects on bio-geochemical properties becomes essential for better risk assessment, mitigation, and examining the fate of arsenic in the ecosystem [44].

### 4.1. Arsenic Speciation in Soil

“Speciation” refers to the presence of an element in various chemical structures, oxidation states, and mineral phases, which collectively account for its cumulative concentration within an ecosystem [63,64]. Understanding chemical speciation becomes very important because bioavailability, physiological impacts, and possible As toxicity largely depends on the specific As species present in the system [65]. Further, speciation helps to better assess a metalloid’s bio-geochemical behavior as compared to its total content [66,67,68], and thus properties like phytoavailability and As uptake by plants are governed by its speciation in soil [69]. This can be explained by the fact that a part of total As that is present in the form of a stable mineral is not bioavailable [32]. Instead, an As species that is otherwise adsorbed on soil constituents may be easily extractable and thus more bioavailable [70]. The various chemical forms in which As may exist in soil include (i) free ionic forms, (ii) precipitated as solids, (iii) adsorbed on soil organic or inorganic constituents, (iv) exchangeable, and (v) structural constituent of primary and secondary minerals [67].

### 4.2. Inorganic and Organic Forms of Arsenic

Arsenic predominantly appears as an inorganic species in natural ecosystems [71]. In water and soil (terrestrial ecosystems), it can be found in four distinct oxidation states: As(-III) (arsine), As(0), As(III) (arsenite), and As(V) (arsenate) [42]. Two principal oxidation states of arsenic i.e., As(III) and As(V), are largely dominant in contaminated soils and sediments and are also the forms in which As enters plant systems [43,53]. Inorganic arsenites and arsenates are also present in the soil in other forms, for instance, the fully protonated arsenous acids or arsenic acids and different oxoanions [72]. For instance, species like HAsO_4_^2−^, H_2_AsO_4_^−^, and H_3_AsO_3_ are the thermodynamically stable species of As(V/III) that have been identified in soil. H_2_AsO_4_^−^ is the most commonly present chemical species in the aerobic soil types [73].

Arsenic may also exist in organic forms in natural environments, the major species being monomethylarsonous acid [MMAA(III)], dimethylarsinous acid [DMAA(III)], monomethylarsonic acid [MMAA(V)], dimethylarsinic acid [DMAA(V)], trimethylarsine (TMA), arsenocholine (AsC), arsenobetaine (AsB), arsenosugars, trimethylarsine oxide (TMAO) and tetramethylarsonium ion [74,75]. Though these species are predominantly found in marine organisms, they have also been identified in small quantities in terrestrial environments [76,77,78]. Microorganisms can methylate the inorganic As to produce methyl As [79,80]. Apart from microbial activity, the use of As-based organic pesticides and insecticides is also responsible for the introduction of organic As in terrestrial environments [61,71]. Under anaerobic conditions, organic As is reduced to volatile arsine, including monomethylarsine [MMA(-III)], dimethylarsine [DMA(-III)], and trimethylarsine [TMA(-III)] [81].

**Figure 1:**
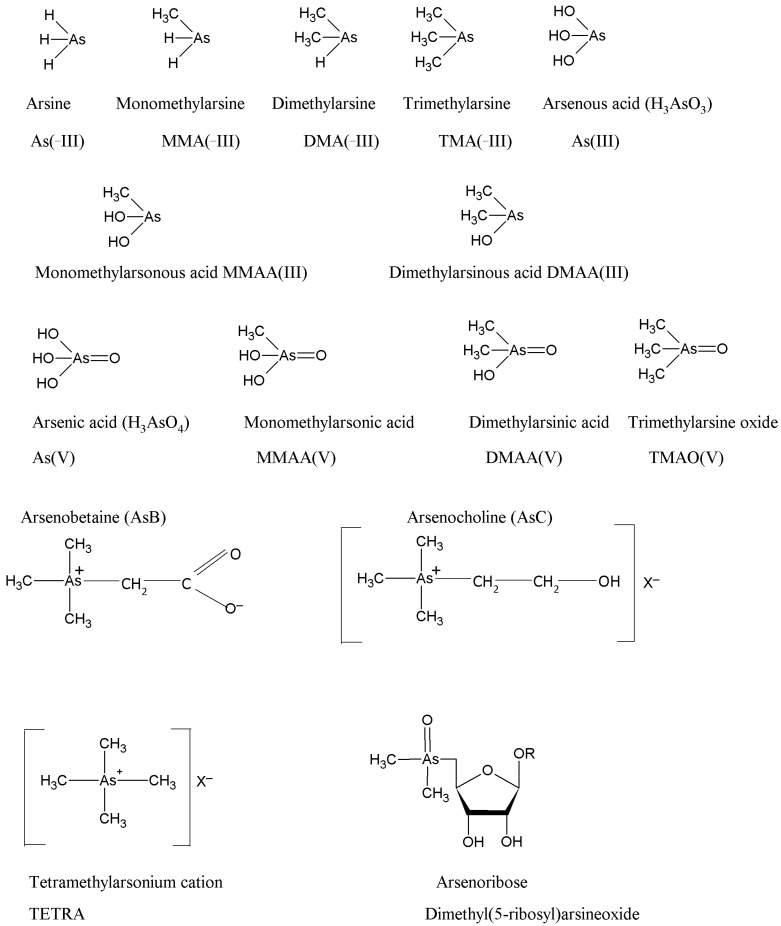
Chemical structures of inorganic and organic As species detected in terrestrial plants. Adapted from [62,71].

Arsenic(III), As(V), MMAA(V), and DMAA(V) are the most commonly present As species in terrestrial ecosystems including plants [60,82]. The molecular structures of some key As species (both inorganic and organic) are indicated in Figure 1.

The presence of As in diverse chemical structures in the plant-soil systems is dynamic, and rapid inter-conversion between them may occur because of redox cycling, methylation of the inorganic forms to organic ones, and/or through the microbial transformation of methylated species of As into the inorganic forms [83,84] depending on the prevailing biotic and abiotic conditions [75,85].

### 4.3. Toxicity Assessment of the Various Arsenic Species

Both inorganic (arsenite and arsenate) and organic As (MMAA(V), DMAA(V), TMAO) can cause toxicity in plants and humans [86,87] though the absorption of MMAA(V) and DMAA(V) is generally lower than the uptake in the form of As(III) and As(V) [88]. The As toxicity to living systems is not only linked to the extent of pollution but also to its speciation in the ambient environment [89,90,91]. Therefore, understanding speciation becomes essential for ascertaining As toxicity. Inorganic As is highly toxic/lethal and more mobile than organic As species [92]. However, it is also reported that dimethylarsenate [DMAA(V)] is more toxic to wheat than inorganic As(V) species [45]. Between the inorganic forms, As(III) is more soluble, more mobile, and also 60 times more toxic than As(V) [32]. Though pentavalent organic As toxicity is not as substantial as As(III) and As(V), trivalent organic As has been found to have higher toxicity than their pentavalent counterparts [93], and thus the presence of MMAA(III) and DMAA(III) in soils and sediments has recently drawn attention [94]. The mechanism of toxicity of arsenite can be understood by its ability to inhibit a protein’s catalytic action and cellular processes by attaching to its sulfhydryl group and inducing structural changes [95]. In contrast, As(V) which is a phosphate analog exercises its toxicity in plants by interfering with phosphorylation and ATP synthesis (key processes of phosphate metabolism).

The toxicity effect of arsenate As(V) is less because it is less bioavailable than As(III). Arsenates being highly stable, are readily adsorbed and retained on oxides/hydroxides of metals, organic matter, and clays in soil, thus rendering them less bioavailable for uptake by plants [44]. On the other hand, weak adsorption of As(III) is an outcome of its neutral nature in soil [96]. However, certain microorganisms can enhance As bioavailability by discharging or converting As into the more water-soluble and mobile As(III) form [97]. Apart from its speciation, As toxicity (due to its accumulation) varies with plant species [32]. For example, in rice, the bioavailability of various As species follows the order As(III) > MMAA(V) > As(V) > DMAA(V) [98]. It was also observed that the translocation rate of organic species of As in rice was greater than inorganic As species [99].

### 4.4. Factors Affecting Arsenic Speciation and Its Bio-Geochemical Properties

The bio-geochemical features of As in soil-plant systems, e.g., mobility, bioavailability/phytoavailability, as well as toxicity, are related to its chemical speciation [100]. The different chemical forms of As (inorganic and organic) are rapidly interchangeable [75] and therefore it becomes imperative to highlight the effect of the speciation of As on its bio-geochemical characteristics. Speciation and As phyto-uptake are affected by processes like precipitation/dissolution, sorption/desorption, oxidation/reduction, redox conditions, As-ligand complex formation, biological transformations, volatilization, plant species diversity, etc. Various other physico-chemical soil properties like pH, Eh (redox potential), clay content, microbial presence, and inorganic oxides of Fe, Al, and Mn also affect As chemical speciation. It is well known that any changes in these physico-chemical and, biological characteristics of soil, or the processes in soil or environmental conditions, can significantly alter speciation, thereby affecting the mobility and bioavailability of As [44].

### 4.5. Soil pH Governs Arsenic Speciation and Bio-Geochemical Properties

An integral factor governing the chemical speciation of As in soil and in turn its biogeochemical properties is the pH of the soil [86,101]. The relative amounts of inorganic As species (arsenite and arsenate) present in the soil vary with changes in soil pH [102]. In acidic soils (especially pH < 5.5), there is an increase in the amount of As(III) (the more soluble form of inorganic As) [103], increasing its phytoavailability. At pH < 2.5, As(V) is totally protonated [104], making its retention by soil particles difficult. Thus, the mobility and bioavailability of As increase as the acidity of the soil increases [105]. Table 2 presents the impact of pH on the chemical speciation of As(III) and As(V).

Under varying pH conditions, arsenate is adsorbed or co-precipitated differently on different metal ions/oxides/constituents present in the soil. While in acidic soils, it is adsorbed on Fe and Al oxides [110], in alkaline soils it co-precipitates over calcium, sulfate, or carbonate ions [111]. This is in coherence with a similar study [112], on the similarity in behavior of As to that of phosphate under different soil pH conditions. It reported Fe-As to be more predominant than Al-As in soils of low pH (around 4), and Ca-As to be significantly present in high pH soils (pH 6–8). Enhanced sorption of As(III) by montmorillonite and kaolinite under pH ranging between 3–9 was reported in a study [113].

### 4.6. Soil Redox Potential (Eh) Governs Arsenic Speciation and Bio-Geochemical Properties

The soil redox potential can substantially affect As speciation, and therefore also its solubility, mobility, and bioavailability [114]. Seasonal variations and changes in groundwater levels are known to vary redox conditions in the soil, and therefore influence its geochemical behavior. The impact of redox conditions is pronounced in As because of the difference in the solubility and mobility of arsenite and arsenate. A general observation is that the solubility of As increases in soil with its decreasing redox potential (Eh value). While As(V) is dominant under oxidized environments, under anoxic conditions (reduced environment), the comparatively insoluble arsenate As(V) is reduced to a more soluble form arsenite As(III) [75,106]. In anaerobic soil, the prevalent As chemical species is H_3_AsO_3_. Given the greater prevalence and ready bioavailability of As(III) in the reducing conditions commonly found in paddy soil, rice plants tend to absorb a higher quantity of this harmful species [115]. Table 3 presents a variation in As speciation with changing redox conditions of soil [44,116].

### 4.7. Other Physico-Chemical Factors That Govern Arsenic Speciation and Bio-Geochemical Properties

Other factors that affect the bio-geochemical behavior of As like mobility, solubility, and bioavailability are discussed briefly here. The presence of organic matter like humic and non-humic compounds in the soil can also alter As speciation by modifying soil conditions. Soils with high organic matter harbor more microorganisms which leads to the production of reducing conditions [117]. Arsenate to arsenite reduction in soils with higher organic matter has been reported in a study [118]. This implies that the organic amendments in the soil increase the bioavailable fractions of As [109]. Studies have also established the effect of the presence of biochar on As bio-geochemical behavior [119]. It has been reported that the biochar addition affects the pH of soil such that it subsequently increases As solubility and mobility, leading to its enhanced bioavailability [111]. Clay content is relatively more efficient than any other parameter of soil for retaining As(V). Arsenic is adsorbed at a higher level in soil with greater clay content [120]. Sorption of As on clay follows the order kaolinite > vermiculite > montmorillonite [121]. In loam and coarse-grained soils, the mobility and bioavailability of As are greater as compared to clay and clay loam soils [122]. Inorganic oxides/hydroxides of Al, Fe, and Mn, if present in soil, act as scavengers of As, and thus decrease their solubility, mobility, bioavailability, and bioaccessibility [123]. Iron/manganese oxides, owing to their high surface area [124], have a great sorption affinity for As. Studies have indicated that soil rich in these inorganic oxides and hydroxides has high As content, and thus Fe and Mn oxides were explored for the remediation and amendment of sites contaminated with As [125]. Arsenic adsorption on inorganic oxides and hydroxides is indirectly affected by soil pH as it modifies the net charge on functional groups of mineral surfaces. The presence of ions like phosphate, calcium, and elements like silicon in the soil also affect As bio-geochemical properties. While phosphate in soil can induce As mobilization and solubilization thereby enhancing its phyto-uptake [37,126], the silicon presence in soil improves the growth of plants by arsenite As(III) exclusion [95] or by activating a plant’s antioxidant systems that help in sequestering As toxicity [127]. The existence of artificial organic ligands like nitrilotriacetic acid (NTA), ethylenediaminetetraacetic acid (EDTA), and glycol ether diaminetetraacetic acid (EDGA) decreases the pH of the soil, thereby increasing the soluble fractions of As(III) in soil [128]. Thus organic ligands increase As solubility and mobilization in soil by decreasing soil pH [103]. On the other hand, cations like calcium tend to immobilize As by interfering and competing with As plant uptake, thus affecting bioavailability [129].

### 4.8. Presence of Microorganisms Affects Speciation and Bio-Geochemical Properties

Besides the physico-chemical features of soil, microorganisms also affect the speciation and bioavailability of As in soil-plant systems by modifying the chemical speciation of As in soil [130]. Because of their ability to interconvert As(III) and As(V), microorganisms can both solubilize and immobilize As in soil-plant systems [116]. Bacterial species like *Agrobacterium tumefaciens*, *Alcaligenes faecalis*, *Bacillus*, and *Geobacillus* can synthesize arsenite oxidase and oxidize As(III) into As(V) [44,131]. Microorganisms such as *Crysiogenes arsenates, Geospirillum barnesi, Bacillus arsenicoselenatis*, and *Desulfutomaculum auripigmetum* act as arsenate-reducing agents, use As(V) in their respiratory processes [44,132] and release As(III) which being the more soluble form of inorganic As causes As mobilization in soil [44,133]. These microbially-induced redox conversions of As between As(V) and As(III) alter its phytoavailability [134]. Inoculation of microbes in soil has also been reported to increase or decrease the phytoavailability of As. For instance, arbuscular mycorrhizal fungi reduced As phytoavailability in maize [135].

## 5. Perception of Arsenic by Plants

Plants obtain carbon/oxygen/nitrogen, minerals, water, and energy to sustain themselves. Heavy metals (HMs) are naturally occurring elements and some of them are essential in minimal quantities in plants to maintain regular functions and include Co, Cu, Mn, Mo, Fe, Mg, Se, Ni, Se, and Zn, but above physiological tolerance levels, they become toxic and induce negative influences on the plants’ growth [136]. However, HMs such as As, Cr, Cd, Pb, Hg, and Ag exhibit deleterious effects even in lesser quantities [137]. Aquaporins, aquaglyceroporins, phosphate, and sulfate transporters in plant systems facilitate arsenic absorption. Since As(V)’s oxyanion chemical structure is physically similar to that of Pi, it rapidly enters plant roots via phosphate (Pi) transporters and is the predominant As species in aerobic soils [138].

## 6. Phyto-Arsenic Uptake

Designing mitigation strategies to address the problems associated with food chain contamination necessitates an understanding of phytoarsenic absorption and its metabolization. Arsenic is present in the ecosystem in different types namely inorganic [arsenate; As(V)], organic [arsenite; As(III)], and methylated derivatives monomethylarsonic acid and dimethylarsinic acid). Among these, As(V) and As(III) are the most familiar types detected in soil and water, with inorganic As being more abundant than organic As. Aerobic soils have a higher concentration of As(V) while under a submerged environment As(III) predominates [88]. Arsenic phyto-uptake is dependent on its overall concentration as well as its speciation in the rhizosphere [52].

### 6.1. Inorganic Arsenate Uptake

Different types of As are absorbed by plant roots through various mechanisms. Arsenic(V) is chemically similar to phosphate (Pi), therefore Pi transporters (PHT) are principally responsible for its transport into most cells. Inorganic phosphate and As(V) share the same transporters since they compete with one another for uptake in a variety of plant species [139]. Various PHTs were detected in plants [140]. Plasma membrane-associated PHT1 transporters primarily engage in Pi absorption and its re-mobilization in plant systems [141]. The number of high-affinity PHT1 family members varies from four in *Pteris vittata* [142], nine AtPHT1 in *Arabidopsis* [143], 11 in *Hordeum vulgare* [144], 13 in *Oryza sativa* [145], 21 in *Triticum aestivum* [144] and 12 in *Setaria italica* [146].

In *Arabidopsis* AtPht1;1, AtPht1;4, AtPht1;5, AtPht1;7, and AtPht1;9 transporters have been shown to express maximally in roots and mediate Pi and As acquisition from the soil into the plant cell [57,147]. In another study, *Arabidopsis* mutant *pht1;1–3* displayed increased As accumulation due to a decline in As(V) uptake. They also observed repression of genes responsible for Pi uptake and induction of genes regulated by As(V) indicating the presence of opposite signaling mechanisms for acquiring Pi and As(V) [148]. Overexpression of the vacuolar phosphate transporter *VPT1* (helps in vacuolar Pi accretion) [149] gene in *Arabidopsis* enhanced the accumulation of As, while *vpt1* mutant plants displayed reduced As accumulation and increased As(V) tolerance. Under a Pi-sufficient environment, expression of *PHT1* genes was observed to be down-regulated in *vpt1*-modified plant types leading to reduced As accumulation [150]. PHT1 transporters also enhance As accumulation in *Salix* spp. [151].

Different investigations have shown the involvement of gene coding for *Oryza sativa* phosphate transporter (*OsPT*/*OsPht1*) in As(V) transport after uptake. Overexpression of rice *OsPT1* resulted in enhanced As(V) accumulation in shoots and roots. In *ospt1* mutant plant roots, the concentration of As(V) was comparable with WT, while in shoots, a 60% decline was observed, indicating that *OsPT1* is participating in the transfer of As(V) from rice roots to shoots [152]. Similarly, scientists also established the role of *OsPT8* in As(V) uptake in rice plants [153] and demonstrated the *OsPT4* gene role in As absorption and transport by analyzing transgenic rice plants harboring its overexpression and CRISPR constructs [154]. *ospt4* mutant plants displayed a decline (20–40%) in As concentration in the xylem sap as well as rice grains when compared to the wild type [155]. The *Oryza sativa NITROGEN LIMITATION ADAPTATION 1* (*OsNLA1*) gene has been shown to facilitate As(V) uptake and tolerance by regulating PHTs [156].

*Pteris vittata*, the first As hyperaccumulator fern characterized, accumulates a higher amount of As due to the presence of high affinity As transporters, PvPht1;3 and PvPht1;4 [157]. PvPht1;3 when expressed in tobacco resulted in enhanced accumulation and translocation of As in shoots [158]. *Panax notoginseng* Pi transporter genes *PnPht1;1*, *PnPht1;2,* and *PnPht1;3* also increase uptake of As(V) from soil [159].

Arsenic(V)-treated rice plants showed a major presence of As(III) species in the xylem sap [160]. Different investigations have shown that As(V) is rapidly converted into As(III) in the roots before xylem loading for translocation into shoots with the help of arsenate reductase (AR) [161]. *PvACR2* (AR gene) of *P. vittata* suppressed the sensitivity of As(V) and As hyperaccumulation when transformed in *Saccharomyces cerevisiae* deficient in the *ScACR2* gene [162]. Overexpression of the *AtHAC1* (High Arsenic Concentration 1) gene in *Arabidopsis* [163] and *OsHAC1;1, OsHAC1;2,* and *OsHAC4* in *Oryza sativa* led to enhanced As efflux, and tolerance, and decreased accumulation of As [164].

### 6.2. Organic As(III) Uptake in Plants

Plants utilize a subfamily of aquaporins (AQP) channels or membrane intrinsic proteins (MIPS) to transport trivalent As across the membranes [165]. Nodulin 26-like intrinsic proteins (NIPs) belonging to AQP are bidirectional transporters that help in As(III) loading into the xylem and also have a role in silicon transport [166]. Studies have also suggested that As(III) transport follows a pathway similar to silicon transport [167]. The plasma membrane intrinsic protein (PIP) and tonoplast intrinsic protein (TIP) subfamily of AQP have also been shown to be involved in As(III) transport [168]. Different NIPs, silicon transporter PIPs, and TIPs responsible for As(III) uptake in plants have been listed in Table 4.

## 7. Arsenic Detoxification

Most metals, including As are detoxified in the vacuoles. Metal binding peptide phytochelatins (PCs) sequester As into vacuole by chelating it via its thiol groups [179]. Two PC transporters of *Arabidopsis thaliana* named AtABCC1 (ATP-binding cassette) and AtABCC2 conferred As(III) endurance by transferring As(III)-PC complex into vacuoles [180]. Similarly, in rice, *Oryza sativa* C-type ATP-binding cassette transporter OsABCC1 has indicated the tendency to detoxify As by sequestering it into companion cells through the vacuoles of phloem, also preventing the transport of As into grain [181].

In another report, *Arsenical Compound Resistance3* (*ACR3*) gene from *P. vittata* was shown to sequester As(III) into vacuoles and confer As tolerance [182].

In *Arabidopsis thaliana*, disturbance of inositol transporters AtINT2 and AtINT4 caused the decline in As(III) concentration in the phloem, seed, as well as siliques. Different experiments concluded that inositol carriers are engaged in loading As(III) in phloem [183].

Rice OsNRAMP1 (Natural Resistance-Associated Macrophage Protein) transporter, when transformed in *Arabidopsis* revealed an enhanced As accumulation. It localized in the plasma membrane of endodermal and pericycle cells, implying its part in the loading of the xylem [184]. In the knockout mutant of rice *osabcc7,* a decline in the concentration of As was observed in the xylem sap and shoot implying its function in As(III) translocation from root to shoot [185]. In another report, a knockout mutant of a rice peptide transporter OsPTR7 inhibited the transport of dimethyarsinate (DMA) from root to shoot. The decreased concentration of DMA in rice grains, indicates its role in translocation [186]. The Rice *OsMATE2* gene, when introduced in tobacco, resulted in a decline in the transportation of As from root to shoot. Rice transgenic plants harboring *OsMATE2* (belonging to multidrug and toxic compound extrusion protein family) RNAi construct (targeting endosperm) displayed a decline in As levels in the rice grain [187]. Recently a member belonging to the glutathione-S-transferase family (tau class), OsGSTU5 conferred As tolerance. Through their experiments, [188] inferred the role of OsGSTU5 in As chelation with GSH followed by sequestration of the complex into the vacuole (root) for detoxification with the help of OsABCC1 transporter hence blocking its transport in the shoot.

**Figure 2:**
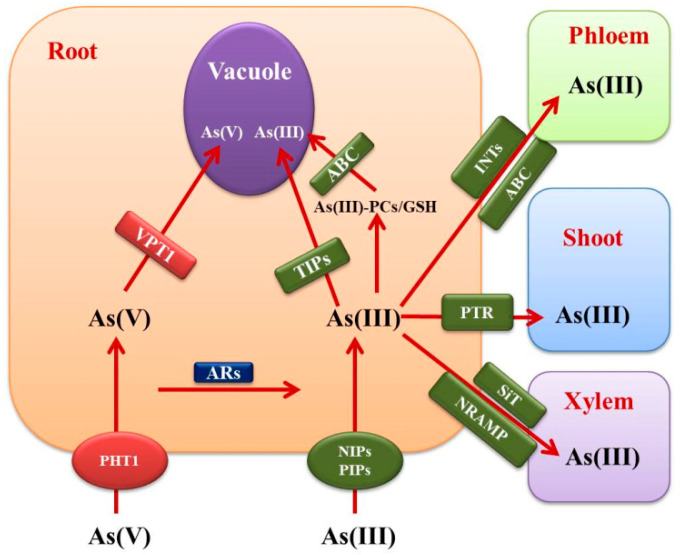
Proposed pathway for phyto-uptake of arsenite and arsenate via different categories of transporters. [ABC: ATP-binding cassette; As(V): Arsenite; As(III): Arsenate; AR: Arsenate reductase; GSH: Glutathione-S-transferase; INTs: Inositol transporters; NIPs: Nodulin 26-like intrinsic proteins; NRAMP: Natural Resistance-Associated Macrophage Protein; PCs: Phytochletins; PHT1: Phosphate transporter; PIPs: Plasma membrane intrinsic proteins; PTR: Peptide transporter; SiT: Silicon transporter; TIPs: Tonoplast intrinsic proteins; VPT1: Vacuolar phosphate transporter].

Based on the literature available, a pathway explaining the vertical transport of As from root and shoot can be proposed (Figure 2). According to that pathway, As(V) is taken up by roots through PHTs, while under reducing environments, roots absorb As in the form of arsenite with the help of proteins belonging to the subfamily of AQPs named NIPs, PIPs, and TIPs. Investigations have also suggested that once it enters the root cells, As(V) is quickly reduced into As(III) by the AR enzyme and is then transported to the xylem with the help of silicon transporters (SiT). In rice, a transporter belonging to the NRAMP family significantly transported As into the xylem. In *Arabidopsis*, inositol transporters are accountable for loading As(III) into the phloem. Finally, As is detoxified in the cell due to its sequestration in vacuoles after getting chelated with GSH or PC. GSH/PC-complexed As is transported to vacuoles through the transporters belonging to the ABC transporter family.

## 8. Impact of Arsenic on Plants

Arsenic induces harmful effects on morphological features such as chlorosis, cell death, disturbances in mineral homeostasis, epinasty, necrosis, reduced biomass, and stunted growth [189,190]. At the subcellular level, As inactivates biomolecules by either blocking essential functional groups or displacing essential metal ions along with ROS [190] and RNS formation. Reduced length of root and shoot and chlorophyll content causes a decline in the net photosynthetic rate in *Vicia faba* seedlings [191], reduced seedling length, biomass, relative water content (RWC), and biochemical constituents in chickpea (*Cicer arietinum* L.) [192], and a down-regulated ascorbate-glutathione cycle, with the lower endogenous level of nitric oxide (NO) in soybean [193] are other effects of As. Elevated levels of MDA, H_2_O_2_, and cell membrane injury are signatures of As stressed plants. Arsenate (AsV) stress led to the induction of apoptosis in *Vicia faba* L. seedlings, the up-regulation of NADPH oxidase and glycolate oxidase activity followed by the downregulation of antioxidative enzymes in leaves 328 [194]. Arsenic was greatly accumulated in roots and shoots, showed root cell death, and a declined endogenous NO level by inhibiting nitric oxide synthase-like (NOS-like) enzyme activity [195], and a decreased uptake of phosphorus resulting in photosynthesis inhibition and cell death in brinjal [195].

### 8.1. Morphological Impact and Response

Arsenic is a poisonous metalloid naturally occurring in soil. Almost all plant species are extremely vulnerable to As contamination in soil, which harms the plant’s growth and development. Some of the physico-chemical properties of As show similarities to phosphorus. It is available in two forms i.e., arsenite As(III) and arsenate As(V). Arsenite is more mobile and more highly toxic than arsenate. It exists in anaerobic soil, while arsenate is found in aerated soil [196]. The toxicity effect varies with different forms of As, soil parameters that control its accumulation in plants, and also the type of plant species. Even a very low As concentration leads to different morphological, biochemical, and physiological variations in the plant system. The plants subjected to As are severely affected and show several As-stimulated symptoms. Roots are prime sites of As exposure and exposure to As reduces its extension and proliferation. From roots, it is translocated to shoots. It also slows down or stops plant growth by arresting cell division and expansion. This also affects the nutrient acquirement and metabolic process in plants. It also results in decreased biomass and reduced fertility of the plants [197]. In plants, the initial effect of As-stimulated toxicity results in a decrease in biomass as well as grain yield. It also reduces germination, leaf area and number, necrosis of margin and tips of leaves, wilting of leaf blades, chlorosis, discoloration, demodulation, and plasmolysis of the cell. It induces leaf senescence and defoliation. It also decreases the production of fruit and the yield of the plant [197].

Seedlings of *Helianthus annuus* L. displayed healthier growth and performed better under the condition of limited exogenous application of As (4 mg/kg soil) [40]. However, at higher concentrations, it inhibits the essential metabolic and biochemical activities that can even cause plant death. A study by [198] demonstrated a substantial plant height reduction with an increased As level in irrigation water. Seedlings of *Cicer arietinum* L. [199] and *Oryza sativa* L. [200] showed stunted roots and shoot growth. As inhibited the leaf number and area, as well as the dry and fresh mass of these plants [201]. The effect of varying concentrations of As (0, 10, 20, 30, and 40 mg/L) was examined on *Trigonella foenum-graecum* L. and *Lathyrus sativus* L. [202] and 11 diverse factors of these two plants were analyzed mainly during the germination and primary seedling growth periods. It was found that the mean value for germination (index, percentage, and relative rate) declined with an expansion in the degree of damage (caused by As) with growing As concentration in both these plants. They also developed a novel Arsenic Response Index (ARI) for crops at the germination and primary seedling growth phase. The impact was substantially higher at 30 and 40 mg/L. Moreover, the dry weight and length of both shoots and roots decreased considerably, particularly at these two concentrations. Tissues showed a substantial accumulation of As and displayed a more severe effect on roots than shoots. *T. foenum-graecum* L. demonstrated an improved tolerance to As-stimulated toxicity compared to *L. sativus* L. For *L. sativus L*., 30 mg/L of As and 40 mg/L for *T. foenum-graecum L*. were considered toxic during germination and primary seedling growth stages [202]. When exposed to As, soybean plants showed a high degree of injury during growth. They also showed decreased root and shoot biomass and low yield [203]. There was a 45 and 30 percent reduction in the biomass and number of pods and beans when the plants were treated with irrigation water containing As (10 mg/L or more) [204]. This injury accounted for oxidative damage and photosynthetic pigment reduction [205]. Soybean plants store As in root tissue primarily by forming complexes with thiol compounds (mostly homo-glutathione-hGSH and homo-phytochelatine-hPC) and via compartmentalization in the vacuole [204]. A low portion of metalloids also accumulates in grains, posing risks to animal and human health in the food chain. In an experiment, soybean plants grown in hydroponic solutions and subjected to high concentrations of As and Se resulted in low biomass, discoloration, and stunted roots. Leaves were small and showed a vertical orientation when compared to untreated plants. Interestingly, plants subjected to Se also demonstrated morphological changes; however, it had less effect on biomass (root and leaf) than As treated plants in comparison to the control [206].

### 8.2. Physiological Impact

#### 8.2.1. Arsenic Stress and Induction of Reactive Oxygen Species

The oxidation state of As, a redox-active metalloid, affects both its mobility and toxicity, with arsenite (As(III)) as highly poisonous and as mobile as arsenate (As(V)) [207]. Oxidative stress induction in plants is one of the foremost responses of As [208]. This oxidative stress results in an overall change in plants’ biochemical and physiological functioning. Additionally, As is shown to directly trigger oxidative stress through the production of ROS at the time of transition of its valence states or implicitly by repressing antioxidant enzymes by attaching to their -SH groups [209]. Within a plant cell, As generates superoxide, hydrogen peroxide, hydroxyl ions, singlet oxygen, peroxyl radical, and hydroperoxyl radical [46]. The ROS are largely produced within mitochondria in plants [210]. Superoxide is produced by mitochondrial complexes I and III in the electron transport cycle [211]. Arsenic exhibits mitochondrial toxicity by impeding succinic dehydrogenase activity [212] and dissociating oxidative phosphorylation along with superoxide generation, which results in the creation of additional ROS [213]. Arsenic also induces the formation of ROS through NAD(P)H oxidase The membrane-bound enzyme NAD(P)H oxidase generates superoxides by transporting electrons from the cell’s internal NAD(P)H around the membrane and uniting those electrons with molecular oxygen to make superoxide anions [214]. Reactive oxygen species can also be produced by As through NOS, a putative enzyme generating NO from arginine and molecular oxygen, bypassing the production of superoxides [215]. Arsenic exposure results in the uncoupling of nitric NOS and arginine, forming ROS [213]. Further, some studies indicate As inhibits NOS activity within the body [216]. The generated oxidative stress and increase in ROS result in the oxidation of lipid molecules in the membranes. Arsenic thus damages lipids, causing the oxidation of cellular membranes, the generation of fatty acid radicals (ROO•), MDA, and HNE, as well as the deactivation of membrane-bound receptors [217]. The peroxidation of membrane lipids induced by ROS also results in the leakage of cellular contents and electrolytes [218]. It is reported with a decrease in the membrane stability index (MSI) of *Pteris ensiformis* in a dose-dependent manner [54]. This instability not only affects the cell membrane but also all the membranes associated with a plant cell including its organelles.

#### 8.2.2. Arsenic Stress and the Photosynthetic System

In plants, As reduces photochemical efficiency, which can have an impact on the light-harvesting complex (LHCII), photosystem II (PSII), as well as chlorophyll and carotenoids [219]. After plants absorb As, the light-harvesting apparatus may be impacted by decreased photosynthetic activity-II and chlorophyll concentrations or by suppressing a few of the essential processes described above. Due to insufficient photosystems-I and -II adaptive modifications brought on by high As levels, a notable decrease in chlorophyll pigment production has been documented [32]. It is also assumed that the Mg atom in the chlorophyll’s tetrapyrrole ring may be replaced with a trivalent As atom, which may also impede the pathway that produces chlorophyll [220]. Additionally, studies on *Ceratophyllum* revealed that some enzymatic stages at the initiation of the tetrapyrrole pathway are affected by As which is evident from the fact that treatment of As even at a low concentration coproporphyrinogen III which is an early precursor of chlorophyll biosynthetic pathway is undetectable [221]. The tetrapyrrole biosynthesis pathway includes an enzyme known as δ -aminolevulinic acid dehydratase (ALAD), which acts as an intermediary. It does this by combining two molecules of δ-aminolevulinic acid (δ-ALA), which results in the formation of the pyrrole, porphobilinogen. Porphobilinogen is an essential plant pigment precursor engaged in photosynthesis, light-sensing, respiration, and the intake of nutrients [222]. It has been observed that As can impede ALAD [223]. δ -aminolevulinic acid dehydratase is a sulphydryl-containing enzyme, and because As has shown a higher affinity for –SH, it forms an effective compound with ALAD, which inhibits the enzyme’s activity. This results in the formation of δ-ALA [224]. A carbon-centered radical (ALA•) and superoxide are produced during the metal-catalyzed autoxidation of delta-ALA at physiological pH. The imino form of delta-ALA is created by further oxidizing the ALA• to produce superoxide, which dismutates into H_2_O_2_ [225]. Additionally, superoxide produced by delta-ALA stimulates the endoplasmic reticulum to release Fe, which may aid in the production of •OH via the Haber–Weiss process [226]. Apart from oxidative stress, As also inhibits photosynthesis by damaging the chloroplast [227]. It is reported from a study on *Azolla filliculoides* that As treatment resulted in a distortion of the shapes of the chloroplast. This was accompanied by the disorganization of thylakoids. In addition, there was breakage of some of the plastids into an amorphous greyish substance along with fragments of thylakoids [228]. Similar results were also obtained from the studies on *Pteris vittata* where chloroplast took a rounded shape accompanied by disintegration of the chloroplast membrane and onset of plasmolysis [229]. All of these result in a decrease in the rates of photosynthesis, transportation of electron and capability to use water [230]. In addition, As stress causes a slowdown in the Calvin cycle and an increase in the level of oxidised NADP+ because only a tiny part of the total carbon is fixed into CO_2._ When ferredoxin is excessively reduced during photosynthetic electron transfer, this oxidised form of NADP+ can act as an electron acceptor. As a result, through a process known as the Mehler reaction, electrons can go from PS-I to O_2_ to create superoxide. This starts a chain reaction that quickly produces oxygen radicals [32].

#### 8.2.3. Arsenic Stress and Respiratory Process

Plant mitochondria are the hub of redox metabolism and are intricately related to different energy metabolism and catabolism pathways [231]. In addition, mitochondria also act as a source of signals in a redox system [232]. A primary source of ROS in mitochondria is the mitochondrial electron transport chain [233]. Quinones, metal cores, and flavin are all involved in electron transport [234]. They can transmit a single electron to molecular oxygen, reduced to superoxide, depending on their redox state and position within a respiratory complex [235]. Complex III (cytochrome b/c1 complex) and Complex I (NADH dehydrogenase) are the two primary sites of superoxide generation [236]. Arsenic can exert its toxicity in the mitochondria in several ways. According to reports, As enters mitochondria through mitochondrial dicarboxylate transporters. These transporters are positioned on the inner mitochondrial membrane and are in charge of dicarboxylate exchange with co-substrates like Pi between the cytosol and the organelle matrix [237]. As a result of As’s inhibition of complex I in the mitochondrial electron transport chain, excessive ROS are produced, causing lipid peroxidation and protein degradation as well as the development of the mitochondrial permeability transition(MPT) [46].

In general, Pi transport channels are used by plants to store and metabolize As(V) [238]. Arsenic(V) shares many similarities with inorganic phosphate molecules [239], which can impair at least phosphate-dependent metabolic processes. During phosphorylation processes, it may compete with phosphate, creating frequently unstable and transient AsV adducts [43]. One such is the uncoupling of photophosphorylation and oxidative phosphorylation that occurs due to synthesis and rapid autohydrolysis of AsV-ADP, which reduces the cell’s capacity to make ATP and perform regular metabolism [240]. In addition, the ATP gamma-phosphate is imitated by arsenate. Competing with phosphate at the active place of ATP synthase prevents the creation of ATP. When the strength of phosphate is low, arsenate is a more potent inhibitor because it prevents both the Pi-H_2_O exchange and the ATP-Pi exchange, which are both mediated by the ATP synthase [241]. By blocking crucial cell metabolic enzymes, As can reduce the lifespan of cells. To inactivate the dihydrolipomide dehydrogenase of the pyruvate dehydrogenase complex, arsenite, a sulfhydryl reactive metalloid, can covalently attach to the vicinal dithiols [242] or sulfhydryl groups of Lipoamide (a cofactor of dihydrolipoyl dehydrogenase enzyme) [243]. As a result, cellular gluconeogenesis and oxidative phosphorylation are compromised. This prevents pyruvate from converting to acetyl coenzyme A (acetyl CoA), which affects the Krebs cycle’s ability to produce ATP [244]. Arsenic also inhibits ketoglutarate dehydrogenase [245].

Regarding the mechanism of the reactions, the enzymes and co-enzymes involved in the oxidative decarboxylation of pyruvate and ketoglutarate are identical. Arsenic interferes with the -SH groups of reduced dihydrolipoamide, which disrupts the conversion of lipoic acid to acetyl-CoA and succinyl-CoA. Arsenic inhibits lipoic acid regeneration by forming a covalent compound with dihydrolipoic acid. In the absence of ATP synthesis, respiration is halted when As prevents the formation of acetyl-CoA and succinyl-CoA and disrupts the TCA cycle [243].

At the time of As toxicity, arsenate ions compete with phosphate ions due to their structural similarity, resulting in pyruvate generation without the net production of ATP. Glyceraldehyde 3-phosphate dehydrogenase (GAPDH) typically oxidizes glyceraldehyde-3-phosphate to 1,3-bisphosphoglycerate in the presence of inorganic phosphate [246], which is then changed to 3-phosphoglycerate in the presence of phosphoglycerate kinase and releases one molecule of ATP [247]. It should be noted that this enzyme can also catalyze the conversion of arsenate and glyceraldehyde-3-phosphate into 1-arseno-3-phosphoglycerate [248], which then hydrolyzes to create arsenate and 3-phosphoglycerate without the production of an ATP molecule [243]. Arsenate and inorganic phosphate compete for GAPDH [249]. This occurrence repeats itself. The loss of ATP synthesis is caused by the released arsenate’s subsequent reaction with glyceraldehyde-3-phosphate. As a result, arsenate decouples oxidative phosphorylation at the mitochondrial level. ADP-arsenate is created when arsenate is present instead of ATP. ADP-arsenate depletes ATP since it hydrolyzes and is not as stable as ATP [243].

#### 8.2.4. Arsenic Stress and Damage of DNA

Arsenic has considerable genotoxic potential and can produce DNA damage such as micronuclei production, aneuploidy, chromosome abnormalities, sister chromatid exchange, deletion mutations, and DNA-protein cross-linking [250]. Nucleosomes serve as the fundamental repeating unit in the packaging of eukaryotic DNA in the form of chromatin [251]. Histones H2A, H2B, H3, and H4 bind to create the histone octamer, which encloses 147 base pairs of DNA [252]. Several post-translational changes can occur, including acetylation and deacetylation, methylation and demethylation, phosphorylation, citrullination, sumoylation, biotinylation, and ubiquitination at the N-terminal end of these histones by the activity of certain enzymes [253]. As a result of these covalent interactions, the structure of chromatin is altered, which in turn stimulates the transcription of genes [254]. Histone acetyl-transferase and histone deacetylase control the alteration of histone acetylation. In this modification procedure, lysine amino acid residue receives an acetyl group transfer in the presence of acetyl coenzyme A, losing its capacity to bind the DNA phosphate groups. The condensed chromatin structure can be opened up by acetylation, which lowers the DNA’s affinity for the histones and releases the histone tails from their attachment to the linker DNA. This makes it possible for transcription factors, co-factors, and RNA polymerase II complexes to access the DNA. The levels of acetylation are determined by the equilibrium between the activities of an enzyme called histone acetyltransferase (HAT) and another enzyme called histone deacetylase (HDAC) [255]. It has been demonstrated that As directly binds to histone acetyltransferase, which results in a decreased level of global histone H4 acetylation at lysine 16 [256]. Arsenic can act on histone modification, namely regulating H3 methylation [257]. DNA methyltransferase, also known as DNMT, is the enzyme responsible for methylating CpG cytosine when there is SAM (S-adenosyl methionine) present to act as a methyl donor [258]. It has also been reported that As can inhibit the DNA activity of methyl transferase either by minimizing its expression levels or directly rendering the enzyme ineffective [259].

### 8.3. Biochemical Impact of Arsenic Stress

When plants undergo stress due to As, the metabolism of essential carbohydrates like sugars and starch is altered detrimentally. Arsenic stress often causes the buildup of soluble sugars in plants [260]. Amylases facilitate the hydrolysis process for internal 1,4-glycosidic bonds in starch to yield low molecular weight products, including maltose, glucose, and maltotriose units [261]. Studies report that As inhibits alpha-amylase activity in wheat in a concentration-dependent fashion [262]. Starch phosphorylase, a plant-based counterpart of α-glucan phosphorylase, is a key player in the metabolism of starch because it catalyzes the reversible conversion of α-1,4-glucan and inorganic phosphate into glucose-1-phosphate [263]. It is observed that Arsenate, which is chemically related to phosphate in terms of size, shape, and charge, inhibits the activity of potato phosphorylase. Arsenate can substitute for phosphate to create glucose 1-arsenate, an extremely fleeting compound that is rapidly hydrolyzed [264]. An experiment reported that As treatment caused a decline in the activity of sucrose synthesizing enzymes, namely sucrose phosphate synthase, whereas sucrose degrading enzymes, namely invertase and sucrose synthase improved in action [265].

Plant development and stress resistance depend heavily on lipid metabolism, which includes lipid production and breakdown. Lipid oxidation, which is thought to be a very destructive process, is a result of As stress. Various species exposed to As stress showed increased cellular electrolyte leakage and damage to cell membranes [266]. It is observed that Lipid molecules in both organelle membranes and cellular undergo peroxidation due to the excessive production of ROS caused by As [225]. Oxidation of membrane lipids results in the production of MDA and hydroxynonenal [267]. Arsenic exposure was also observed to alter the lipid biosynthesis pathway. In rice, comparative transcriptome analysis revealed that treatment with As(III) dramatically altered the expression of 59 genes linked to lipid production [268]. Arsenic could influence lipid signaling by inducing the phosphatidic acid signal in addition to lipid peroxidation and biosynthesis [205]. One of the most significant signal molecules in plants, phosphatidic acid is a crucial precursor in the manufacture of triacylglycerols and glycerophopsholipids [265].

The increased affinity of inorganic As for sulfhydryl groups in proteins causes cellular death and membrane damage in plants, making it a potent disruptor of plant metabolism. It has been observed that plants’ total protein content decreases in response to As exposure [220]. Arsenic stress results in the reduction of activities of nitrate assimilatory enzymes, namely nitrate reductase, nitrite reductase, and glutamine synthetase in rice plants [269]. Proteases and peptidases are the enzymes responsible for hydrolyzing proteins into their constituent amino acids [270]. It has been observed that in As-treated rice seedlings, there was a decrease in activities of RNAase and protease which resulted in impairment of hydrolysis of RNA and protein [271]. In addition, As-induced oxidative stress is likely to oxidize proteins resulting in the formation of protein carbonyls [272].

## 9. Mechanism Undertaken to Alleviate Arsenic-Induced Stress

### 9.1. Arsenic Immobilization and Compartmentalization

Arsenic is generally taken up in the inorganic form in collaboration with transporter proteins [53] which is dependent on the speciation of the metal [101]. Such trace metals are usually deposited in the cytoplasm, cell wall, and vacuole, and the detoxification is governed by selective exclusion of metals, metal retention, enhancement of the enzymatic system for triggering tolerance, immobilization by the cell wall, and extracellular carbohydrates, formation of complexes by binding with phytochelatins which are peptides of low-molecular-weight or by ligands, organic acids and amino acids, and lastly by compartmentalization [273]. Arsenic and phosphorus (P) are chemically analogous to arsenate (As (V) [32], and As (V) uses several Pi channels to pass in the plant cells. Different Pi transporter proteins (PHT) support As (V) uptake by the cell and are unidirectional [57], whereas, As(III) uptake involves various nodulin-26-like intrinsic proteins (NIPs) aquaporin channels and is bidirectional [32] which uses Si transporters because it shows similarities with it [274,275]. The Pi transporters *AtPht1;1* and *AtPht1;7* are sensitive to As(V) [57]. WRKY transcription factors like *WRKY6* and *WRKY45* modulate the expression of *AtPht1;1* which helps in As (V) uptake [276]. *Pht1* transporter, *PvPht1;3* in *Pteris vittata* has shown a greater affinity towards As(V) by repressing the gene activation involved in Pi starvation [148]. The nodulin-26-like intrinsic proteins (NIPs) aquaporins like *NIP2;1, NIP5;1, NIP6;1, OsNIP3;3, HvNIP1;2,* and *NIP3;1* mediate the As(III) transportation [173]. Due to tetrahedral size and similar Pka values (9.2 for arsenious acid and 9.3 for silicic acid), a silicon transporter Lsi1 also promotes As(III) transportation in plant cells through Casparian strips of root tissues [167]. Another aquaporin tonoplast intrinsic protein (TIP), *PvTIP4;1* is involved in As(III) uptake in *Pteris vittata* [277]. Overexpression of inositol transporter genes *AtINT2* or *AtINT4* helps in As(III) loading in phloem [183].

Methylated arsenic (DMA and MMA) uptake mainly occurs from the xylem and phloem, thereby reaching the grain [278], which uses a glycerol transporter and aquaglyceroporins for its transportation [279]. The uptake of As in cytosol occurs by the formation of complexes with phytochelatins and is sequestered into the vacuole through cross-membrane transporters present in the vacuole tonoplast [180]. The binding of As to the plant cell wall is possible because of the configuration of the cell wall which comprises polysaccharides, amino acids, proteins, and phenolics which helps in the accumulation of the heavy metal [280]. The number of polysaccharides with carboxyl groups present in the cell wall decides the attachment capability of trivalent and divalent heavy metals which are reported as alginates in algae [281] and homogalacturonans (HGA) in higher plants [280]. Pectin is the major polysaccharide of the plant cell walls and comprises four domains namely homogalacturonans (HGA), rhamnogalacturonan I (RGI), rhamnogalacturonan II (RGII), and xylogalacturonan (XGA). It constitutes 30% of type I primary walls in dicots whereas, secondary and type II primary walls in monocots [282]. Among the four pectin domains, homogalacturonans (HGA) are produced from the Golgi apparatus and translocated to the cell wall [280]. The homogalacturonans are demethylesterified by pectin methylesterase, which causes the cell wall’s loosening, increasing the porosity and allowing permeability of As into the plant cell [283]. The pectin methylesterase not only adds methanol and proton in pectin, causing the synthesis of negatively charged carboxyl groups but also triggers gel formation, enhancing the As binding in the pectin matrix [284].

Several proposals for the internal detoxification of As are established by immobilization and compartmentalization [285]. The plant cells have a storage compartment called the vacuole, which shows blockades of HM ions, especially As. It was proved that vacuolar compartmentalization of As occurs in *Pteris vittata* due to the accumulation of more than 90% of As in the protoplast which was translocated in the vacuole as it conjugates with the organic acids and low molecular weighted thiolic peptides known as phytochelatins [286]. These complexes endure stability and re-oxidation to arsenite is prevented because of the acidic pH in the vacuole which causes compartmentalization of As in the vacuole [58]. An As(III) efflux protein ACR3 (PvACR3) which is confined at the tonoplast of *P. vittata* gametophytes increases the transportation and sequestration of As in vacuoles [287]. The enzyme arsenate reductase assists in the reduction of As(V) to As(III) after the uptake, which thereby makes a pathway for the formation of As(III)-phytochelatin complex and transports it across the tonoplast [288] by two ABC transporters AtABCC1 and AtABCC2 localized in the vacuole [180]. Loading of arsenite into the xylem was controlled by a gene encoding Lsi2, an efflux protein [289]. This efflux protein generally transports both As(III) and Si(IV) [288]. Similarly, transportation of As to grains is possible by phloem loading, where As(III) cannot form a complex with the phytochelatins due to the neutral pH of the phloem sap despite its higher concentration [290]. So, As(III) accumulation is found in ovular vascular traces [9]. Arsenic(V) uses Pi transporter for intercellular movements. It is found to be a co-substrate of three mitochondrial dicarboxylate transporters, proteins which are found to be restricted to the inner mitochondrial membrane and are liable for dicarboxylate interchange with co-substrates such as Pi, between the cytosol and the organelle matrix [237].

**Figure 3:**
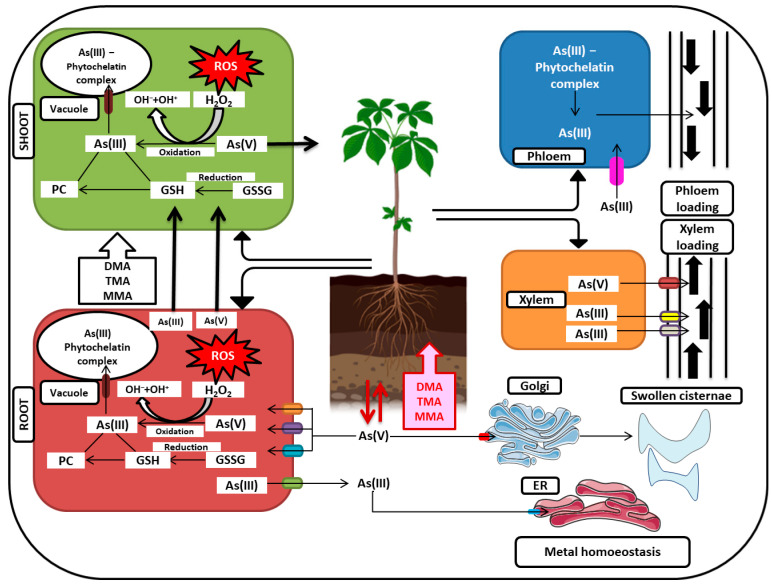
Diagrammatic representation of arsenic immobilization and compartmentalization in different organelles of the plant cell.

Inhibition of the metal trafficking process and its prevention from plant cell entry occurs by sequestration through apoplast [291]. The influx transporters are associated with the uptake of heavy metals into the cytosol, whereas efflux transporters are involved in the compartmentalization of the metals into the organelles [292]. An aquaglyceroporin, AtNIP1 transports As(III) and localizes it to the endoplasmic reticulum [293] nip1;1ko mutant helps compartmentalize the As(III), confirming a role in metal homeostasis on the membranes of the endoplasmic reticulum, NIP1;1 regulates detoxification in the vacuole by interacting with SNARE SYP51 [291]. The Golgi apparatus is another organelle where the metals are sequestered through secretory trafficking pathways directly from the cytosol by the presence of CDF antiporters in the Golgi apparatus’s membranes [294]. The mobility of dictyosomes associated with the alteration of cytoskeletons of the Golgi apparatus is disrupted during the metal stress. The cisternae become swollen and the Golgi becomes disorganized [291]. Vacuolar trafficking and autophagy are connected to the plasticity of the endoplasmic reticulum (ER) and Golgi apparatus (GA) and are involved in the compartmentalization of the metal in Multi Vesicular Bodies which are connected with ER and GA [295]. These are designated as pro-vacuoles and are separate from general vacuoles which contain different sets of transporters for the compartmentalization of the metals in their tonoplast [291]. Figure 3 depicts the diagrammatic representation of As immobilization and compartmentalization in various organelles of the cell for better understanding.

### 9.2. Triggering of Antioxidant Defense Responses

Enhanced ROS production and metal stress responses in plants have been extensively studied [296]. Besides modified phosphate breakdown, oxidative stress is a different mechanism through which As toxicity manifests in plants [297]. Arsenic generates ROS by inhibiting critical enzyme systems and electron leakage during the conversion of As (V) to As(III). The reduction of inorganic As is followed by methylation, which is a redox-driven event, and such outcomes could produce ROS [298]. Biomethylation of As produces monomethyl arsonic acid (MMA), dimethyl arsenic acid (DMA), tetramethyl alarsonium ion (TETRA), and trimethylarsenium oxide (TMAO). Arseno-choline, arsenobetaine, and arseno-sugars are a few other forms of metabolized As that have been identified in plants [299]. One way that plants can cope with As-induced stress is by activating their antioxidant defense systems. These systems help to neutralize harmful free radicals that are created due to As exposure. Some ways that plants can trigger their antioxidant defense responses include:

#### 9.2.1. Upregulation of the Activity of Antioxidant Enzymes

Plants can increase the production of enzymes such as superoxide dismutase (SOD), catalase (CAT), and peroxidase (POD). These enzymes help to remove harmful free radicals and reduce oxidative stress.

- Superoxide dismutase (SOD)

It is a category of metalloisozymes that convert the extremely reactive superoxide radical into oxygen and hydrogen peroxide, hence playing a crucial role in protecting stressed cells [300]. Superoxide dismutase is a metal cofactor-associated antioxidant enzyme. Cu/Zn-SOD resides in the cytosol, peroxisome, plastid, and root nodules, whereas Mn-SOD resides in the mitochondria and Fe-SOD resides in the plastids. *Holcus lanatus*, red clover, and Mung bean exposed to As exhibit increased SOD activity and decreased lipid peroxidation [301,302]. During As stress, the upregulation of Cu/Zn SOD in rice seedlings has been documented [303]. Analysis of native page SOD activity reveals an increase in the capability of one Mn-SOD and two major Cu/Zn SOD isozymes in arsenate-exposed red clover [302]. Copper/Zinc SOD is one of the highly responsive enzymes to As involved in cellular homeostasis during redox disturbance, as shown by the proteomic study of maize root [304]. It is reported that SOD activity increased in response to a low As concentration, whereas a high As concentration inhibits the accumulation of SOD mRNA and causes its activity to decrease [305]. In reaction to arsenate, genes that code for SOD and peroxidase play a key role. In response to As (V) stress, the microarray experiment indicated the increase of chloroplast Cu/Zn SOD (at2g28190), Cu/Zn SOD (at1g08830), and a SOD Cu chaperone (at1g12520), as well as the severe inhibition of FeSOD [306].

- Catalase

Catalase is a tetrameric, heme-containing enzyme that may decompose H_2_O_2_. It is found in the peroxisomes, glyoxisomes, cytosol, mitochondria, and root nodules. It degrades hydrogen peroxide swiftly into the water and molecular oxygen without utilizing cellular reducing equivalents. Therefore, CAT protects the cell by removing hydrogen peroxide using an energy-efficient approach. In As-tolerant Chinese brake fern (*Pteris vittata*), CAT activity is greater than in As-sensitive thin brake fern (*Pteris ensiformis*) and Boston fern (*Nephrolepis exaltata*) [297]. CAT activity increased in *Zea mays* when exposed to As [305]. In contrast to the abovementioned findings, As-induced decreases in CAT activity have also been seen in Mung bean and *Taxithelium nepalense* [301].

- Ascorbate Peroxidase

In the chloroplast, the dismutation of superoxide radicals by the SOD enzyme releases H_2_O_2_. To avoid inhibition of Calvin cycle enzymes, further H_2_O_2_ removal or detoxification is required [307]. Due to the absence of CAT in chloroplasts, plants have adapted an alternate route to the peroxidase–ascorbate–glutathione pathway to detoxify hydrogen peroxide. Peroxidase requires a reductant to convert hydrogen peroxide to water; in plant cells, ascorbate serves as this agent [308]. APX is a protein in the plastid stroma and membrane containing heme. In the presence of APX and two molecules of ascorbate, hydrogen peroxide is converted to water, and two molecules of monohydroascorbate are produced [309]. Rice seedlings [127], Mung beans [301], Beans [310], and Maize [311] exposed to As exhibited increased APX activity.

- Glutathione Reductase

It is responsible for maintaining glutathione (GSH) levels for most cellular processes [312]. It resides in the mitochondria, cytosol, and plastids. GSH is oxidized to glutathione disulfide (GSSG), which is necessary for the regeneration of ascorbate from monodehydroascorbate. Glutathione reductase catalyzes the NADPH-dependent transformation of GSSG to GSH. Few studies have examined the behavior of GR during As-induced oxidative stress in higher plants. The activation of GR demonstrated the increased need for glutathione (GSH) during As-induced oxidative stress in rice seedlings [303]. Although elevated GR activity has been detected in the roots of *Pteris vittata*, *Pteris ensiformis*, and *Nephrolepis exaltata*, GR activity in fronds and rhizome is greater in As-hyperaccumulator *P. ensiformis* and *N. exaltata* than in As-hyperaccumulator *P. vittata* [297].

- Glutathione S-transferase

The enzymes generated by toxic metals and oxidative stress are omnipresent, multifunctional enzymes that utilize the tripeptide glutathione (GSH) as a co-substrate or coenzyme [313]. Six functional types of this enzyme were identified: phi, tau, theta, lambda, zeta, and DHARs. GSTs are dimeric proteins [313]. Among the catalytic actions depending on GSH are the conjugation and subsequent detoxification of cytotoxic compounds. Arsenic is reported to stimulate the production of GST in mesquite and maize plants [314].

#### 9.2.2. Increasing the Synthesis of Non-Enzymatic Antioxidants

Plants can also produce non-enzymatic antioxidants such as ascorbic acid (vitamin C) and glutathione. These molecules help to scavenge free radicals and safeguard plant cells from harm. Ascorbic acid is known as one of the most plentiful antioxidants in plant cell stroma, chloroplast, apoplast, cytosol, and vacuole. The Ascorbate (AsA) pool in chloroplast includes a substantial amount of reduced ascorbate to safeguard photosynthetic components. The ascorbate-glutathione cycle plays a significant part in eliminating hydrogen peroxide and regeneration of membrane-bound carotenoids and -tocopherol. It can directly react by decreasing superoxide, hydrogen peroxide, and the hydroxyl radical or by quenching singlet oxygen [54]. Ascorbate (reduced) is also a cofactor of the ascorbate peroxidase (APX) enzyme, which generates dehydroascorbate (oxidized). Ascorbate (reduced) is recycled to dehydroascorbate (DAsA) via a GSH-dependent process catalyzed by dehydroascorbate reductase, which utilizes NADPH as a reducing equivalent. Components of the ascorbate-glutathione cycle have been identified in the cytoplasm, mitochondria, and peroxisome, where it functions as an antioxidant defense system [309].

Regarding the reaction of ascorbate during As-mediated oxidative stress, there are very few publications accessible. It was reported that there is a substantial rise in reduced ascorbate after As exposure, in the fronds of As-hyperaccumulator *Pteris vittata* compared to As-sensitive *P. ensiformis* [54]. The ascorbate concentration increased in the hypocotyls but reduced in the roots of cucumber plants exposed to As [315].

Furthermore, arsenate and arsenite have been found to exhibit a high affinity to thiols like glutathione [316]. Arsenate reductase catalyzes arsenate reduction to arsenite and is also considered a detoxifying process because arsenite can bind to phytochelatins. Arsenate reduction is connected to NADPH oxidation via glutathione reductase’s reduction of oxidized glutathione, with glutathione (GSH) serving as the electron donor for arsenate reductase [162]. It was also observed that GSH functions as a reducing potential during the As reduction in As-stressed bacteria [317]. Through a reaction catalyzed by glutathione-S-transferases, GSH can directly bind to ROS and detoxify it (GSTs). Arsenic exposure has been proven to stimulate glutathione-S-transferase in *Zea mays* [305]. Few studies have examined As’s influence on plants’ glutathione levels. It was further discovered that a higher ascorbate-glutathione pool protects against oxidative damage in the arsenate-tolerant As hyperaccumulator *P. vittata* [54]. Other data implies that rapid arsenate influx led to the depletion of glutathione and the formation of phytochelatins in *Holcus lanatus* [58].

Similarly, the glutathione level of red clover plants treated with 50 mg/kg As decreased [302]. Tolerant plants like *Hydrilla verticillata* exhibit a considerable rise in GSH and PCs in response to As exposure [318]. Moreover, treatment with GSH and cystine eliminated oxidative stress and restored the development of As-exposed rice seedlings [303]. Plant glutathione-S-transferase (GST), which is triggered by toxic metals and oxidative stress, is a ubiquitous enzyme that performs various functions using the tripeptide glutathione (GSH) as a co-substrate or coenzyme. Among glutathione–s transferase’s GSH-dependent catalytic actions are the conjugation and subsequent detoxification of cytotoxic compounds.

#### 9.2.3. Modulation of the Activation of Signaling Pathways

Plants can also activate signaling pathways in response to As stress. For example, the jasmonate signaling pathway has been shown to activate antioxidant defense responses in plants exposed to As. Plant hormones play major roles in forming signaling networks that regulate plant growth and stress-related responses. Jasmonic acid (3-oxo-2′-cis-pentenyl-cyclopentane-1-acetic acid, abbreviated as JA) is an endogenous substance in higher plants that regulates growth. JA, its methyl ester (MeJA), and its isoleucine conjugate (JA-Ile) are fatty acid derivatives known together as jasmonates (JAs). Initially identified as a stress-related hormone, JAs also regulate critical growth and development processes [319]. Arsenic is a redox-active metalloid whose toxicity and mobility strongly depend on its oxidation state, with arsenite (As(III)) being more toxic and mobile than arsenate (As(V)). Humic substances (HS) are also redox-active and can potentially react with As and change its redox state. It is also shown that semiquinone radicals produced during the microbial or chemical reduction of an HS model quinone (AQDS, 9,10-anthraquinone-2,6-disulfonic acid) are strong oxidants [320]. They oxidize arsenite to arsenate, thus decreasing As toxicity and mobility. This reaction depends strongly on pH with more arsenite (up to 67.3%) being oxidized at pH 11 compared to pH 7 (12.6% oxidation) and pH 3 (0.5% oxidation). In addition to As(III) oxidation by semiquinone radicals, hydroquinones that were also produced during quinone reduction reduced As(V) to As(III) at neutral and acidic pH values (less than 12%) [320] but not at alkaline pH. To understand redox reactions between arsenite/arsenate and reduced/oxidized HS, we quantified the radical content in reduced quinone solutions and constructed Eh-pH diagrams that explain the observed redox reactions [207]. The results from this study can be used to better predict the fate of As in the environment and potentially explain the occurrence of oxidized As(V) in anoxic environments. Overall, activating antioxidant defense responses can help plants cope with As-induced stress and reduce the negative effects of As on plant growth and development [321].

### 9.3. Cross Talk between Nitric oxide and Arsenic

To overcome As stress, plants have evolved diverse strategies such as detoxification, metal ions chelation in the cytosol, compartmentalization in organelles (like vacuoles), repairing damaged biomolecules and exporting from the cytosol to other places by use of transporters, and induction of antioxidant enzyme systems including bioactive, gaseous, signaling molecule NO. It is now well established in plant systems that both non-enzymatic and enzymatic systems work for NO generation [322] and are involved in oxidative-nitrosative stress acting as a signaling molecule. NO induces diverse regulatory and morpho-physiological responses in plants such as flowering [323], senescence [324], root development [325], pollen tube growth [326], gene transcription regulation involving posttranscriptional modifications (S-nitrosylation) [327], second messenger generation, mobilization and consequent gene expression [328], programmed cell death [329], seed germination, stomatal movements [330] and seed dormancy [331].

**Figure 4:**
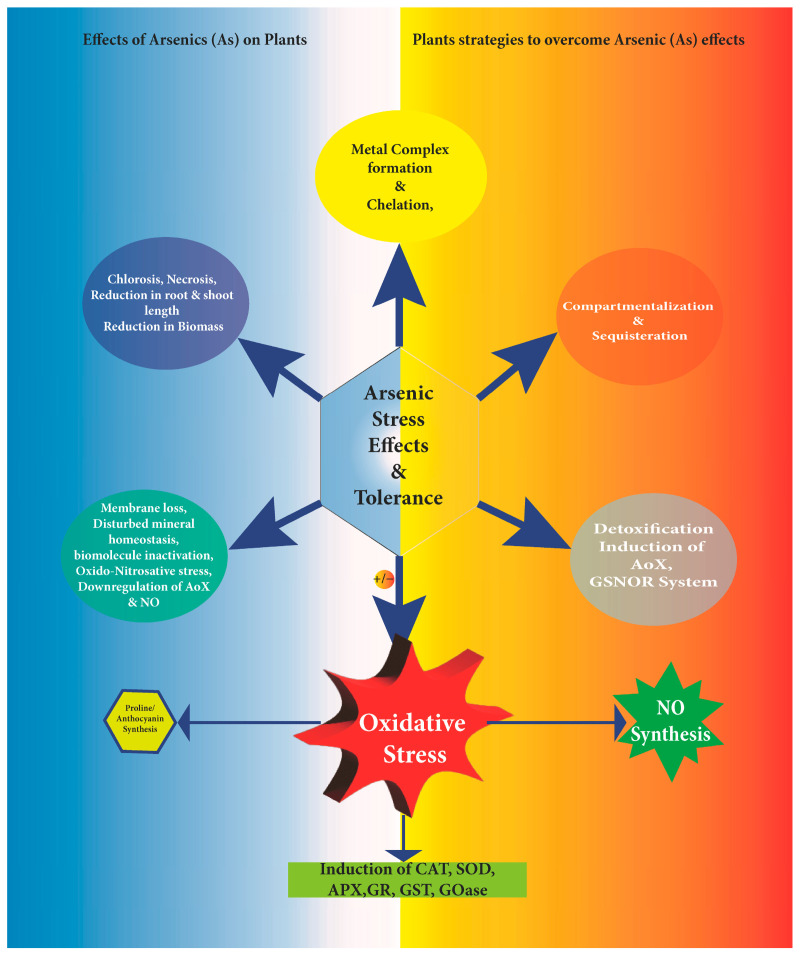
General effects and mechanism of Arsenic tolerance in plants.

The oscillating NO generation pattern and signaling are observed during As stress conditions that help induce the associated antioxidant system for overcoming stress [332] causing an increase in NO content, protein tyrosine nitration, S-nitrosoglutathione reduction (GSNOR) activity with a concomitant reduction in the content of glutathione and S-nitroso glutathione [333]. Both Cd and As uptake gets reduced by NO without affecting the pollutant’s translocation-capability from roots to shoots but contradictory reports are also available that show NO reduces the Cd-induced but not the As-induced oxidative stress by triggering peroxy-nitrite production [334]. Nitric oxide was able to modulate metal transporters such as ABC, Fe transporters, NIP, NRAMP, and stress-related genes (CytP450, GSTs, GRXs, TFs, amino acids, hormone(s), genes related with signaling and secondary metabolism during As stress assisting in As detoxification in *Cicer* sp. [335] and rice by affecting the stomatal density and size, chlorophyll content and consequent increase in photosynthetic rate. Nitric oxide exposure also enhanced lignin content in the root, GSH/GSSG ratio and Pc/As content; decreased arsenite level (As(V)), maintained the antioxidant level, and modulation of several hormones (GA, IAA, SA, JA) along with amino acid content and phenolic metabolite to overcome As-induced oxidative stress [336]. NO and auxin work together to alleviate the As stress by enhancing AUX1 expression, and lateral root (LR) production in the rice root system [334]. Arsenite led to the downregulation of *OsLsi1* and *OsLsi2*, influenced thiol metabolism, enhanced PCs level, and reduced GSH content and GSH/GSSG ratio which was reversed by NO supplementation. Similarly, Fe deficiency in the shoot was reversed by supplementation by affecting the level of Fe transporters gene expression (OsYSL2, OsFRDL1, OsIRT1, and OsIRO2) in rice [337]. Figure 4 illustrates the general effect of arsenic on plants.

Arsenic stress induced the downregulation of polar auxin transporter (PIN proteins) gene expression and genes related to nutrient transport (AMT, NRT, NiR, PHT, KTP). Such changes are reversed by IAA application along with root growth improvement, less As accumulation and sequestering in the vacuole, and improved redox status of glutathione. These events collectively protect from oxidative stress and cell death. Similar responses seen under As + NO treatment [338] suggested that IAA-mediated mitigation is associated with endogenous H_2_O_2_ which might act downstream in brinjal root [195], further suggested in soybean that H_2_O_2_ might have acted downstream of NO ameliorating arsenate toxicity [193]. Exogenous NO application along with SA effectively reversed oxidative stress, the reduction of As content in leaves and roots, and the concomitant enhancement of the antioxidant defense system (AsA-GSH enzymes, GOase enzymes), photosynthetic traits leading to improvement in overall plant growth suggesting NO’s role in SA-induced As stress tolerance in maize plants [339]. Exogenous NO through NO donor and spermidine (SPD) also modulated glyoxalase enzymes and regulated many biochemical pathways to overcome arsenate stress in chickpea (*Cicer arietinum* L.) [192]. P-mediated NO helped to scavenge ROS and methylglyoxal content, photosynthesis restoration, and reduced translocation of As from roots to leaves, which was cross verified by the application of cPTIO (NO quencher) and SNP suggesting the role of P-mediated NO in As stress tolerance [340]. Arsenite toxicity in brinjal root was found to be mitigated also by ASC and GSH independently but was dependent on endogenous NO [195].

### 9.4. Arsenic Mitigation Measures—A Step towards Good Health, Food Security and Sustainable Development

Arsenic contamination in air, soil, and water above permissible limits causes health hazards and requires immediate action. There are various measures/strategies available that can be adopted as human practices, motivating people to abandon old water-extraction practices, planning, and execution for region-specific, need-based, or demand-based approaches. These can be supplemented by ensuring linkage among different stakeholders and subsidy, aid, or tax benefits by the government provided for adopting sophisticated treatment technologies, and these have been thoroughly reviewed [341,342]. The following mitigation measures have been adopted as region-specific or in general as per availability, financial support, technical know-how, adaptability behavior of people, proactive actions of governments, or international support:

#### 9.4.1. Simple, Semi-Efficient but Cost-Effective Human Behavior-Based Measures

Such methods intend to ensure minimum or arsenic-free water supply to the community. These include raising awareness, the marking of wells with high arsenic concentrations, finding alternative water sources by well switching to nearby low As content wells, the use of hand pumps, using sand filters, and adopting rainwater harvesting and solar disinfection [341]. Apart from these, adopting water-conservative methods and differential irrigation methods can also contribute to minimizing As problems. The problems associated with behavior-based measures are highly variable and dependent upon awareness both economic and educational, environmental conditions like hot weather and the desert, the proactive nature of people, and malnutrition [343]. Similarly, irrigation and soil amendment methods are good to mitigate the problem of As but are not fool-proof measures in preventing the entry of arsenic due to the shared essential nutrient transport system.

#### 9.4.2. Simple, Effective but Costly Measures

Although some of the measures described above are good at a local level such as well switching, they require greater work to carry water to the consumption location. Therefore, local measures are adopted that are a little costly but effective such as groundwater extraction from deep tube wells, dug wells, surface water, and rainwater purification for drinking [344].

#### 9.4.3. Adopting Decision-Making Assisting Tools

Computer-based tools help in cost-effective decision making to utilize arsenic-free water. The modeling software (GMS 10.2) which helps to predict both water flow and As transport [345], allows the assessment of potential As content in water bodies and deep and shallow aquifers. Such modeling software along with advanced numerical modeling may assist in better monitoring and health management by avoiding contaminated water use in drinking and irrigation.

#### 9.4.4. Arsenic Elimination Techniques

Arsenic is eliminated from contaminated water by various chemical, biochemical, and biological methods such as oxidation and reduction, adsorption, coagulation/membrane filtration and purification, ion exchange, reverse osmosis, coprecipitation, and microorganism-based oxidation. The toxic nature of As(V) is more than As(III) and is difficult to remove from water. It is solved by the oxidation of As followed by its adsorption on solid surfaces [346,347]. Mn dioxide-polished sand is used as an agent that shows both oxidant and adsorption properties. Similarly, iron oxides, Cu, and Mn ternary metal oxide with nano-adsorbent properties has been developed for effective As removal [348]. Chemicals such as chlorine, copper oxide, H_2_O_2_, KMnO_4,_ and H_2_O_2_ are used as oxidizing agents for As removal [349]. Electromagnetic radiation such as UV radiation is found to be effective in increasing As(III) oxidation in the presence of oxygen due to the generation of hydroxyl radicals through photolysis of Fe(OH)_2_^+^ [350]. The use of additives has also shown a promising effect in controlling As problems. Increasing the availability of other minerals such as Fe-based additives improves free Fe oxide availability in soil and reduces the release of As. Mn oxide diminishes arsenic mobilization by As(III) oxidation and that reduces its uptake. Silicon-containing fertilizer shares a similar uptake pathway as arsenic [167,351]. There are various bacteria such as *Gallionella ferruginea* and *Leptothrix ochracea* known for Fe- and Mn-oxidizing properties that also show effective As removal and are called biological oxidants [352]. Rice fields inoculated with algae and microbial fuel cells have also been used as other biological or bioremediation agents as As removal strategies [353,354]. Biotechnology-based advancement made in the bioremediation of As has been thoroughly reviewed [355].

#### 9.4.5. Nanotechnology-Based Augmentation of As Tolerance in Plants

Nanoparticles (NPs) are tiny molecules (<100 nm diameter) with an enhanced surface area with unique properties. These NPs are manufactured for industrial and agricultural uses, for example, zinc oxide nanoparticles (ZnONPs) and silver oxide nanoparticles (AgONPs). It was found that ZnONPs and potassium (K^+^) positively mitigated As toxicity in Vicia faba L. seedlings by enhancing NO content in normal and As-polluted soil [191]. Zirconium dioxide (ZrO_2_) nanoparticles show high porosity as well as adsorption capacity for As(III) and As(V), therefore can be employed for As mitigation as they can bind both As(III) and As(V) and eliminate As by oxidation of As(III) [356].

#### 9.4.6. NO Based As Stress Tolerance in Plants

Nitric oxide is a bioactive gaseous signaling molecule synthesized by diverse sources and acts as a cytotoxic as well as a cytoprotective molecule depending upon cellular redox status. Nitric oxide works by alleviation of As stress by altering transporter expression, upregulating the antioxidant gene, modifying defense compounds, and the immobilization of As. Simultaneous application of the NO donor and As partially or fully reversed the abnormalities induced by As in *V. faba* roots [357] and alleviated AS(III) induced inhibition by altering nutrients, amino acids, and auxin redistribution via nitrogen and auxin transporter (PIN gene) in *B. juncea* seedlings [358]. However, NO or/and Si alleviated As-induced oxidative stress upregulating antioxidant enzymes such as SOD, APX, GR, GST, GSH, and thiol and proline synthesis which improved growth, gas attributes, and decreased As uptake [340]. Arsenic stress mitigation strategy also involves transporter expression regulation, defense-related genes, root cell wall composition modification, and biosynthesis of enriched sulfur compounds such as phytochelatins (PCs) in *V. faba* [359]. NO also immobilized As in the roots and reduced the shoot translocation by upregulating the transcriptional expression of the PCS, GSH1, MT2, and ABC1, modulated chlorophyll and proline metabolism, increased NO accumulation and stomatal conductance along with crosstalk between the antioxidant related enzyme activity as well as glyoxalase I and II leading to reduced endogenous H_2_O_2_ and MG and enhanced PCs and glutathione accumulation helped protect photosynthetic apparatus in tomato (*Solanum lycopersicum* L.) [360], and in ridged *Luffa acutangula* (L.) Roxb. seedlings [361].

#### 9.4.7. Gene Editing for Developing As-Tolerant Plants Using Molecular and Traditional Plant-Breeding Techniques

Plants generally absorb As(V), the stable form of As, through transporters present in the root cells. The selection of alternative phosphate transporters, low arsenic uptake, and high arsenic resistant properties in plants are key traits to develop As tolerance. It has been found that overexpression of Arabidopsis ABC-type transporters provides high As tolerance [180]. Plants tolerant to As convert As(V) into As(III) in the cytosol, rendering them inactive by the channelization of them to vacuoles. Cereals and other crop plants accumulate As in their edible parts, consequently posing a severe threat to human health. Thus, it is essential to reduce the toxicity induced by As accumulation. Breeding methods have been used to generate As tolerant plants; however, these methods are cumbersome and time-consuming. Biotechnological approaches serve as a promising tool to create transgenics susceptible to As to overcome yield loss and achieve sustainability and food security. Targeting the crucial processes and gene-encoding signaling proteins can help control its build-up in grains. These consist of the uptake of As; sequestration and efflux of AsIII; reduction of AsV; and the methylation and volatilization of As [362]. Disruption of Lsi1 and 2 negatively regulates the AsIII uptake in the root and loading in the xylem in rice [363]. Lsi1 mutants displayed a significantly low strength of AsIII in roots and straw than the wild type (WT), while no substantial difference was observed in grain tissue. On the contrary, Lsi2 mutants showed a considerable drop of AsIII in straw by 13–19% and grain (51–63%) than WT plants [363]. Rice mutant defective in OsPHF1 (Phosphate transporter traffic facilitator 1) showed the loss of function to absorb and transport As (V) from roots to shoots [238]. OsABCC1 (C-type-ATP-binding cassette transporter), identified in rice, is localized in tonoplast and restricts the transport of As to the grains through the sequestration of AsPC complex in the vacuoles of phloem companion cells [181]. Arabidopsis transgenics overexpressing OsGSTL2 showed tolerance to various abiotic stresses, including As stress [364]. OsPT1 regulates the increase of As in rice. T-DNA mutants of OsPT1 (ospt1) exhibited a low total As assimilation in shoots, and no significant change was observed in roots. In contrast, overexpression transgenics demonstrated an increased level of As in shoots and roots than WT [152]. Moreover, ospt8 mutants exhibited less AsV uptake in rice, while mutants ospt8 (obtained by ethyl methanesulphonate mutation) showed less AsV uptake in the root (33–57%) and an enhanced tolerance to AsV than WT plants [153]. A study reported that miR528 negatively regulates the phenotype associated with As tolerance in rice [149]. The miR528 overexpression transgenics were extremely sensitive to As(III) stress. Arabidopsis plants lacking AtABCC1 and AtABCC2 showed high sensitivity to As [180]. In another study, has1 and has2 rice mutants were isolated that showed a higher level of As content than WT. Further, using map-based cloning, OsABCC1 and OsPCS1 genes were identified for has1 and has2 phenotypes correspondingly. This indicated that OsPCS1 and OsABCC1 mutually function in the sequestration of As. OsPCS1 overexpression transgenic showed less accumulation of As in grain [365]. Rice and *Arabidopsis* transgenics overexpressing ACR3 derived from yeast improved the efflux of As(III). Accumulation of As significantly lessened in rice grains [366]. Arabidopsis transgenics overexpressing PvACR3;1 (derived from *Pteris vittata*) exhibited germination even at 80 μM As(III) and 1200 μM As(V) of concentration. The transgenics also exhibited less As accumulation in roots at 150 μM As(V) concentration [367]. OsHAC1;1 and OsHAC1;2 as As(V) reductase was also identified in a study that limits As accumulation in shoots and grains tissue. Overexpression of OsHAC1;1 and OsHAC1;2 decreased As accumulation and improved tolerance to arsenate. The transgenics grown in paddy soils contaminated with AsV displayed a reduction in As by 20% in grain than WT because of enhanced efflux of AsIII [164]. Tobacco transgenics overexpressing PvACR3 showed less assimilation of As(III) in shoots i.e., 22% in comparison to control plants. The transgenics were found to efficiently sequester the excess of As(III) in the root cell vacuoles [368]. Tobacco transgenics overexpressing the ACR2 gene from Arabidopsis offered tolerance to As(V) at 200 μM concentration [369]. The PvACR3;1 gene that encodes As(III) transporter was expressed in Pteris vittata in a study. Accumulation of total As in unhusked grains of rice transgenics expressing PvACR3;1 declined by 28–39% than WT subjected to soils polluted with As. The improved As retention in transgenic roots lowered its translocation in shoots and accumulation in plant parts [367].

In another investigation, HAC4, an As(V) reductase in rice plants was identified [370]. Knockdown (*OsHAC4*) resulted in As(V) reduction in the rice roots and As(III) efflux, thus causing a greater amount of As in shoots. On the contrary, *OsHAC4* overexpression led to better tolerance to As(V) and diminished accumulation of As in shoots and roots of transgenic plants. *OsCLT1* (CRT-like transporter 1) plays a vital role in the homeostasis of GSH by facilitating the export of glutathione and γ-glutamylcysteine from plastids to the cytoplasm and thus regulates detoxification in rice [371]. It was also demonstrated that restricting the transport of phosphate via OsPHO1;2 (phosphate transporter) in addition to overexpression of OsPCS1 (PC synthase) and increasing PC level can facilitate in reducing the accumulation of As in As-tolerant cultivar of rice [372]. Gene silencing of OsPCS using endosperm-specific intron-containing hairpin RNA molecules resulted in the reduction of As level in transgenic rice by 35% in comparison to control WT plants [373]. Indian mustard transgenics overexpressing *AtPCS1* derived from *Arabidopsis* provided tolerance to As and Cd [374]. *Arabidopsis* overexpressing *PvGRX5* (a glutaredoxin gene) obtained from *P. vittata*, (hyperaccumulator of As) conferred tolerance to As(V) via reduction of As(V) and controlling the cellular level of As [375]. Overexpressing *OsGrx_C2.1* and *OsGrx_C7* in *Arabidopsis* conferred tolerance to As and transgenics showed altered expression of *AtNIPs* that led to less As(III) uptake [376]. In rice R2R3-MYB transcription factor, *ARM1* (ARSENITERESPONSIVE MYB1) regulates the transport of As(III). Overexpression of *OsARM1* showed high sensitivity to As(III), while its knockout conferred tolerance to As(III) stress in rice transgenics [377]. *OsWRKY28* regulates As(V) and Pi assimilation in rice and its knockout led to a decline in the accumulation of As and phosphate in the shoots [378]. The expression of OsPRX38, a class III POD from rice, was upregulated manyfold in response to both AsV and AsIII stresses. The overexpression of OsPRX38 significantly improved As tolerance of *Arabidopsis* transgenics by upregulating SOD and PRX GST activity; less electrolyte leakage; and a low level of H_2_O_2_ and MDA. The transgenic displayed better growth as observed by their increase in total biomass and production of seeds than WT under As stress [379]. Rice transgenics overexpressing *OsGrx C7* showed a decline in the accumulation of total As in grains and augments tolerance to AsIII. Total As was less by 67% in unpolished transgenic rice and by 85% in polished rice in comparison to WT [380]. Overexpression of *OsWNK9 Arabidopsis* exhibited enhanced tolerance to arsenite and showed improved biomass, more proline content, and high activities of antioxidant enzymes than WT [381]. Increased AtGolS1 expression and galactinol accumulation in Arabidopsis thaliana under As stress was also reported in a study. Overexpression transgenics of AtGolS1 displayed better growth under As stress, however, *Arabidopsis* mutants were found to be sensitive to As which further suggested AtGolS1 plays a role in overcoming As stress [382].

Various gene editing tools like CRISPR/Cas9; zinc-finger nucleases and transcription activator-like effector nucleases can be used for precise targeting of genes such as *Lsi1/2* and *Pht1:8* that can lessen the uptake of As without affecting P and Si absorption [383]. Effective implementation of genetic engineering necessitates an in-depth understanding of the complex mechanisms of accumulation and sequestration of As, its translocation through the vascular system, phytovolatilization of As, reduction of As(V), and tolerance to As stress [383]. The biotechnological approach and traditional agricultural practices can help decrease As water and soil levels and their further deposition in plants, especially in seeds that pose a severe threat to bio-safety. Thus together, these methods can help in attaining sustainability and food security.

## 10. Prospects and Conclusions

Arsenic, a metalloid, is considered a dominant environmental pollutant and causes severe health issues for living creatures, including humans, making it a local and global concern. Arsenic exists both as an inorganic and organic contaminant with different oxidation states, making it highly reactive and mobile, which determines its bioavailability and toxicity. Soil pH, redox potential, solubility, and microbial activity are other factors that determine the physio-biochemical properties of As. These factors help with the perception of As, its uptake, transport, and distribution in vivo as well as ex vivo, governing its accumulation, detoxification, and homeostasis in plant cells and tissues. Arsenic induces adverse effects in plants ranging from morpho-physio-biochemical levels that eventually cause a decline in expansion, development, and productivity. Tolerant plant species have evolved defense mechanisms such as immobilization, compartmentalization by using phytochelatins and thiols, upregulation of antioxidant levels and non-enzymatic antioxidants, and modulation of several signaling pathways including NO by involving hormones, and other signaling, and bioactive molecules. Researchers are currently aiming to develop eco-friendly technologies like phytoremediation along with augmenting the plants by exogenous supplementation of chemicals like NO donors, silicon, spermidine, phosphates, salicylic acid, potassium, and zinc oxide-based nanoparticles that aid in alleviating the noxious outcomes of As. The research is also aiming to use known plant species to clean up the lands contaminated by As with great emphasis on making it available at a low cost with optimum potential. Now, there is a great need to identify and screen more plant species with excellent phytoremediation potential for As, which will be supported through government policies, programs, and plans to adopt such low-cost and eco-friendly technologies for a massive environmental cleanup. On the other hand, there is also a need to more deeply explore a physio-biochemical and genetic understanding of developing species having the capacity to lessen As uptake or greater plant biomass which in turn can augment a higher capacity to accumulate, detoxify, compartmentalize, or neutralize As to make the environment As free by preventing entry into the food chain thereby ensuring qualitative food security that can contribute to achieving the sustainable development.

## Figures and Tables

**Table 1:** Physio-Chemical Properties of Arsenic.

S.No.	Characteristic Feature	Arsenic
1.	Atomic Number	33
2.	Atomic Weight	74.9
3.	Specific Gravity	5.73
4.	Melting Point	817 °C (at 28 atm),
5.	Boiling Point	613 °C,
6.	Vapor Pressure	1 mm Hg at 372 °C
7.	Nature	Brittle Crystalline Solid

(Source: [34,35]).

**Table 2:** Arsenic speciation in soils with different pH.

Oxidation State of Arsenic	Species Present	pH	Reference
As(III)	H_3_AsO_3_	At pH 0–9, the most dominant form (53–100%)	[44]
	H_2_AsO_3_^−^ HAsO_3_^2−^	pH > 9	
As(V)	H_3_AsO_4_	pH 1–3	[44,106]
	H_2_AsO_4_^−^	pH 3–6	
	HAsO_4_^2−^	pH 7–11	
	AsO_4_^3−^	pH 12–14	
As(V)	H_2_AsO_4_^−^ HAsO_4_^2−^	pH 4–9	[107,108]
As(V)	H_2_AsO_4_^−^ (96%)	pH 3–4	[109]
	HAsO_4_^2−^ (73%)	pH 6–7	

The dissociation constant (pK_1_) value of H_3_AsO_3_ (As(III)) is 9.2 implying it remains undissociated at neutral pH. H_3_AsO_4_ (As(V)) has a dissociation constant pK_1_ = 2.2 indicating it is anionic at neutral pH [81].

**Table 3:** Arsenic speciation in soils with different redox potential.

Redox Condition	Reducing Conditions Low Eh Values −200 to 0 mV	Moderately Reducing Conditions Intermediate Eh Value 0 to 100 mV	Oxidizing Conditions High Eh Value 100–200 mV
Arsenic speciation	As(V) is reduced to As(III)	Partial dissolution of As(V)	As(V) predominates and is co-precipitated with iron and manganese oxides
Reference	[44,85]		

The main redox couples that can facilitate the redox inter-conversion between arsenite and arsenate are Mn(IV)/Mn(II), O_2_/H_2_O, NO_3_^−^/NO_2_^−^, ferric/ferrous, NO_3_^−^/N_2_, CO_2_/CH_4_, and SO_4_^2−^/HS^−^ [44].

**Table 4:** Plant transporter responsible for phyto-uptake of Arsenite.

Category of Transporter	Name of the Transporter	Plant Source	Function	Reference
Nodulin 26-like intrinsic proteins	OsNIP2;1/OsLsi1	*Oryza sativa*	Enhanced As(III) uptake	[167]
	OsNIP2;2/OsLsi6		As(III) transport	[167]
	OsNIP1;1 and OsNIP3;3		As(III) loading in xylem and root-shoot translocation	[169]
	OsNIP3;2		As(III) uptake	[170]
	LjNIP5;1 and LjNIP6;1	*Lotus japonicas*	As(III) transport	[171]
	AtNIP5;1 and AtNIP6;1	*Arabidopsis thaliana*	As(III) transport	[171]
	AtNIP1;1, AtNIP1;2		As(III) uptake	[172]
	AtNIP3;1		As(III) uptake and root-shoot translocation	[173]
	AtNIP7;1		As(III) uptake	[174]
	HvNIP1;2	*Hordeum vulgare*	As(III) transport	[175]
-	DvNip1	*Dittrichia viscosa*	As(III) tolerance	[176]
Silicon transporter	OsLsi2	*Oryza sativa*	As(III) uptake	[167]
Plasma membrane intrinsic proteins	OsPIP2;4, OsPIP2;6, and OsPIP2;7	*Oryza sativa*	Bi-directional permease	[177]
	AtPIP2;2	*Arabidopsis thaliana*	As(III) efflux	[178]
Tonoplast intrinsic protein	PvTIP4;1	*Pteris vittate*	As(III) uptake	[168]

## Data Availability

The data is contained within the manuscript.
